# Dynamic Oligomerization of Integrase Orchestrates HIV Nuclear Entry

**DOI:** 10.1038/srep36485

**Published:** 2016-11-10

**Authors:** Doortje Borrenberghs, Lieve Dirix, Flore De Wit, Susana Rocha, Jolien Blokken, Stéphanie De Houwer, Rik Gijsbers, Frauke Christ, Johan Hofkens, Jelle Hendrix, Zeger Debyser

**Affiliations:** 1 Laboratory for Molecular Virology and Gene Therapy, Department of Pharmaceutical and Pharmacological Sciences, KU Leuven, Leuven, 3000, Belgium; 2 Laboratory for Photochemistry and Spectroscopy, Molecular Imaging and Photonics, Department of Chemistry, KU Leuven, Heverlee, 3001, Belgium; 3 Laboratory for Viral Vector Technology and Gene Therapy, Department of Pharmaceutical and Pharmacological Sciences, KU Leuven, Leuven, 3000, Belgium

## Abstract

Nuclear entry is a selective, dynamic process granting the HIV-1 pre-integration complex (PIC) access to the chromatin. Classical analysis of nuclear entry of heterogeneous viral particles only yields averaged information. We now have employed single-virus fluorescence methods to follow the fate of single viral pre-integration complexes (PICs) during infection by visualizing HIV-1 integrase (IN). Nuclear entry is associated with a reduction in the number of IN molecules in the complexes while the interaction with LEDGF/p75 enhances IN oligomerization in the nucleus. Addition of LEDGINs, small molecule inhibitors of the IN-LEDGF/p75 interaction, during virus production, prematurely stabilizes a higher-order IN multimeric state, resulting in stable IN multimers resistant to a reduction in IN content and defective for nuclear entry. This suggests that a stringent size restriction determines nuclear pore entry. Taken together, this work demonstrates the power of single-virus imaging providing crucial insights in HIV replication and enabling mechanism-of-action studies.

Although active nuclear import is a hallmark in the replication cycle of lentiviruses such as the human immunodeficiency virus type 1 (HIV-1), nuclear entry is one of the least understood steps^1^,^2^,^3^,^4^. After reverse transcription of the viral RNA into double stranded DNA, the pre-integration complex (PIC) is formed as an assembly of the viral DNA (vDNA) and cellular and viral proteins. Prior to integration, the PIC has to cross the natural barrier of the nuclear membrane through nuclear pore complexes (NPCs) which serve as selective entry gates^5^. Recent evidence suggests that uncoating of the HIV capsid (CA) core occurs close to the nuclear membrane although some CA molecules may accompany the PIC into the nucleus^6^,^7^,^8^,^9^. Genome-wide siRNA screens identified the nucleoporins Nup153 and Nup358 (RANBP2) as host cofactors of HIV nuclear import^10^,^11^,^12^,^13^. Nup358 binds CA^14^ and is believed to act as a docking station for the HIV PIC^10^,^14^. Nup153 is located in the nuclear basket; interactions between its FG repeats and either viral integrase (IN) or CA are in line with a role during nuclear entry^10^,^15^,^16^. Besides nucleoporins, importin α/β, importin 7 and Transportin-SR2 (TRN-SR2, TNPO3) have been proposed to be involved in nuclear import of the PIC^1^,^17^,^18^,^19^,^20^. A role for the HIV DNA flap in nuclear import has been proposed as well^21^,^22^.

HIV-1 IN mediates the insertion of the viral cDNA in two consecutive steps: 3′ processing and strand transfer^23^. IN catalytic activity is highly dependent on a dynamic equilibrium of IN multimers; evidence indicates that 3′ processing requires at least a dimer whereas at least a tetramer is needed for concerted integration^24^,^25^,^26^,^27^,^28^. In line with this, the prototype foamy virus (PFV) intasome has been shown to consist of an IN tetramer^29^. Concerted integration of the HIV cDNA occurs into active transcription sites^30^,^31^ and is guided by the host factor LEDGF/p75^32^,^33^,^34^. LEDGF/p75 contains an N-terminal chromatin/DNA binding moiety (residues 1–325) and a C-terminal integrase binding domain (IBD, residues 347–429)^35^,^36^. The pivotal role of LEDGF/p75 in HIV-1 replication was revealed via mutagenesis, RNAi-mediated depletion, transdominant overexpression of the IBD of LEDGF/p75 and cellular knockout studies^32^,^33^,^37^,^38^,^39^,^40^,^41^,^42^,^43^.

Structure-based drug design gave rise to 2-(tert-butoxy)-2-substituted acetic acid derivatives, which bind to the LEDGF/p75 binding pocket at the IN dimer interface and block HIV replication^44^. Although compounds with different structures have been described, they all bind to the same pocket, and are therefore called LEDGINs. LEDGINs have a dual mechanism-of-action, inhibiting the LEDGF/p75-IN interaction and enhancing IN multimerization^45^,^46^,^47^,^48^. More recently, LEDGINs were found to affect late stage HIV replication as well. The phenotype requires binding of LEDGINs to the LEDGF/p75 binding pocket on IN^49^,^50^ and is caused by enhanced multimerization of IN in the virions resulting in morphological defects as evidenced by electron microscopy^49^,^51^,^52^,^53^.

While pools of HIV-1 particles are highly heterogeneous, studies of HIV nuclear entry are typically limited to population-averaged information. Here we performed single virus analysis to reveal the fate of single PICs, in particular their IN content and oligomeric state, during their journey into the nucleus. We employed HIV viral particles carrying fluorescent IN^54^ and two complementary microscopy approaches: 3D confocal microscopy and single-molecule Förster resonance energy transfer (FRET). Nuclear entry is associated with a reduction in the number of IN molecules in the PIC and upon nuclear entry the interaction with the host factor LEDGF/p75 increases IN oligomerization. Addition of LEDGINs during virus production prematurely enhances IN oligomerization in the virion, resulting in stable multimeric complexes in the cytoplasm that are defective for nuclear entry. This argues for a stringent size selection of the HIV IN complex for nuclear entry to occur.

## Results

### Single-virus analysis probes IN content and state

**Figure 1:**
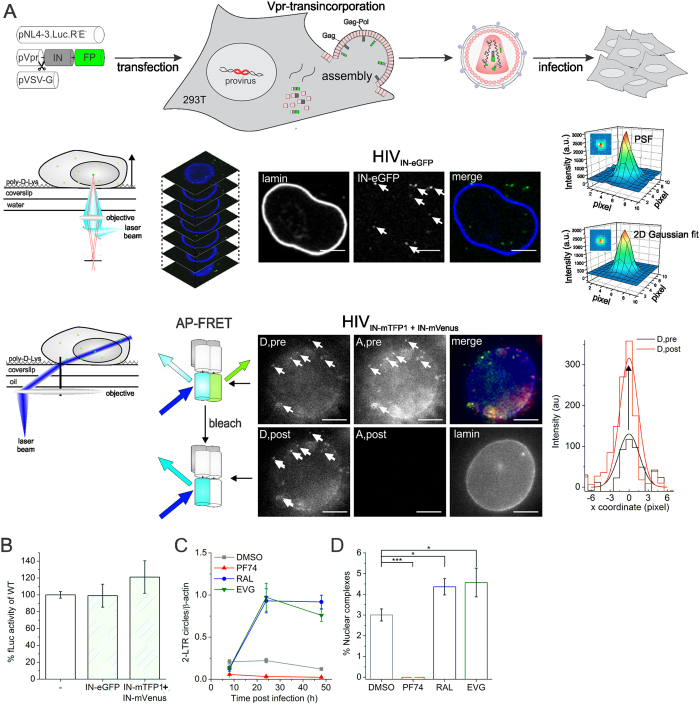
Single virus analysis probes IN content and conformation in functional viruses. (**A**) Top: HeLaP4 cells are infected with VSV-G pseudotyped IN-labeled virus, produced via Vpr-transincorporation of IN-FP, for 6 hours (scissors: HIV protease recognition site). Middle: A 3D confocal image of an HIV_IN-eGFP_ (green dots) infected HeLaP4 cell is acquired. The point spread function (PSF) of each IN-eGFP complex is fitted with a 2D Gaussian function to determine the fluorescence intensity, hence the quantity of IN-eGFP per complex. Scale bars: 5 μm. Bottom: Quasi-TIRFM imaging of HIV_IN-mTFP1+IN-mVenus_ in infected cells enables acceptor photobleaching (AP)-FRET measurements. The intensity of the donor (mTFP1; D, pre), quenched by the proximal acceptor (mVenus; A,pre), is dequenched after photobleaching of the acceptor (mVenus; A, post) and hence its brightness increases (mTFP1; D, post). The fluorescence intensity of each viral complex in both donor images (D, pre and D, post) is used to quantify the mean FRET ratio, as a measure for the interaction between IN subunits. Scale bars: 5 μm. The 2D Gaussian function shows an increased fluorescence intensity of the donor (D, post) after acceptor photobleaching compared to the intensity before photobleaching (D, pre). (**B**) Single round infectivity of HIV, HIV_IN-eGFP_ and HIV_IN-mTFP1+IN-mVenus_ on HeLaP4 as measured by Firefly luciferase (fLuc) activity. Viruses are produced as in (**A**), the Vpr-transincorporated IN-FP is shown and ‘−’ represents the absence of transincorporation. The mean relative luminescence units (RLU) of two experiments performed in triplicate, normalized for protein content of cell lysates is shown, error bars represent standard deviation. (**C**) qPCR analysis for the kinetics of 2-LTR circle formation in HeLaP4 cells after infection with HIV in the presence of DMSO, PF74 (10 μM), RAL (0.6 μM) or EVG (0.2 μM) at 8, 24 and 48 h post infection. Error bars represent standard deviation of triplicates. (**D**) Percentage nuclear IN-eGFP complexes of cells treated with DMSO, PF74 (10 μM), RAL (0.6 μM) or EVG (0.2 μM) during infection with HIV_IN-eGFP_. Error bars represent standard error of mean (SEM); *p-value < 0.05, ***p-value < 0.001 obtained with a Mann-Whitney test.

To investigate the fate of HIV IN during nuclear entry we generated single (or dual-) color fluorescently labeled lentiviral vectors by transfecting 293T producer cells with three (or four) plasmids, one (or two) of which encoding Vpr-IN-FP. IN is fused to a fluorescent protein (FP) and is actively targeted to assembling viral particles by viral protein R (Vpr), which is confirmed by Western blotting (Fig. 1A; Top and Supplementary Fig. S1). In contrast to the viral particles used in previous studies^49^,^53^,^54^, we now retained the wild-type (WT) IN sequence (and not the catalysis defective D64E mutant^53^,^55^) in the packaging construct, resulting in viral particles that maintain 100% of the WT single-round infectivity (Fig. 1B). Confocal laser scanning microscopy (CLSM) was used to quantify the amount of IN-eGFP molecules present in a single viral complex (Fig. 1A; Middle). An advanced image analysis tool was developed, allowing automated detection of each fluorescent viral complex (HIV_IN-eGFP_), determination of their nuclear or cytoplasmic location and calculation of their fluorescence intensity (Supplementary Fig. S2). The intensity of each IN-eGFP viral complex was calculated by fitting the point spread function (PSF) of each spot with a 2D Gaussian function, providing information about the number of labeled IN molecules per complex. Image acquisition and intensity calculations were carefully optimized and validated (Supplementary Fig. S3 and Supplementary Table S1). Quasi-total internal reflection fluorescence microscopy (quasi-TIRFM) was used for single-particle Förster resonance energy transfer (FRET)-analysis to quantify the oligomeric state of IN inside viral complexes (HIV_IN-mTFP1+IN-mVenus_; Fig. 1A; Bottom). First, the FRET donor (“D, pre”; mTFP1) and acceptor (“A, pre”; mVenus) are imaged at low laser power. Subsequently, mVenus is photobleached at high laser power (“A, post”) followed by second imaging of the donor at low laser power (“D, post”). Based on the obtained fluorescence intensity of the fluorescent virions localized in both donor images (“D, pre” and “D, post”) a per-viral complex FRET ratio (Eq. 1) is calculated. If FRET occurs, the mean FRET ratio will be larger than unity. Changes in FRET ratio reflect changes in the interaction between different labeled IN subunits, resulting from altered stoichiometry, oligomerization affinity and/or conformation.

Validation of the functionality of the modified viruses was done using specific HIV-1 replication inhibitors. First, we used the small molecule inhibitor PF74^56^, which binds viral CA and affects reverse transcription (RT), uncoating and nuclear import in a dose dependent manner^6^,^7^,^56^,^57^. In agreement with the reported decrease in 2-LTR circle formation (Fig. 1C)^6^,^56^, a nearly complete block in nuclear import was observed upon treatment with 10 μM PF74 (Fig. 1D). Secondly, treatment with an integrase strand transfer inhibitor (INSTI), either raltegravir (RAL) or elvitegravir (EVG)^58^,^59^ resulted in PIC accumulation in the nucleus (Fig. 1D). This correlates with the increase in 2-LTR circles induced by INSTIs (Fig. 1C)^60^ and corroborates the existence of a fully functional intasome in at least a subset of the complexes. In summary, single-particle analysis of the fluorescent HIV viruses provides a valuable tool for assessing the IN content and oligomeric/conformational state of viral complexes during infection.

### IN content and oligomerization state change in viral complexes upon nuclear entry

**Figure 2:**
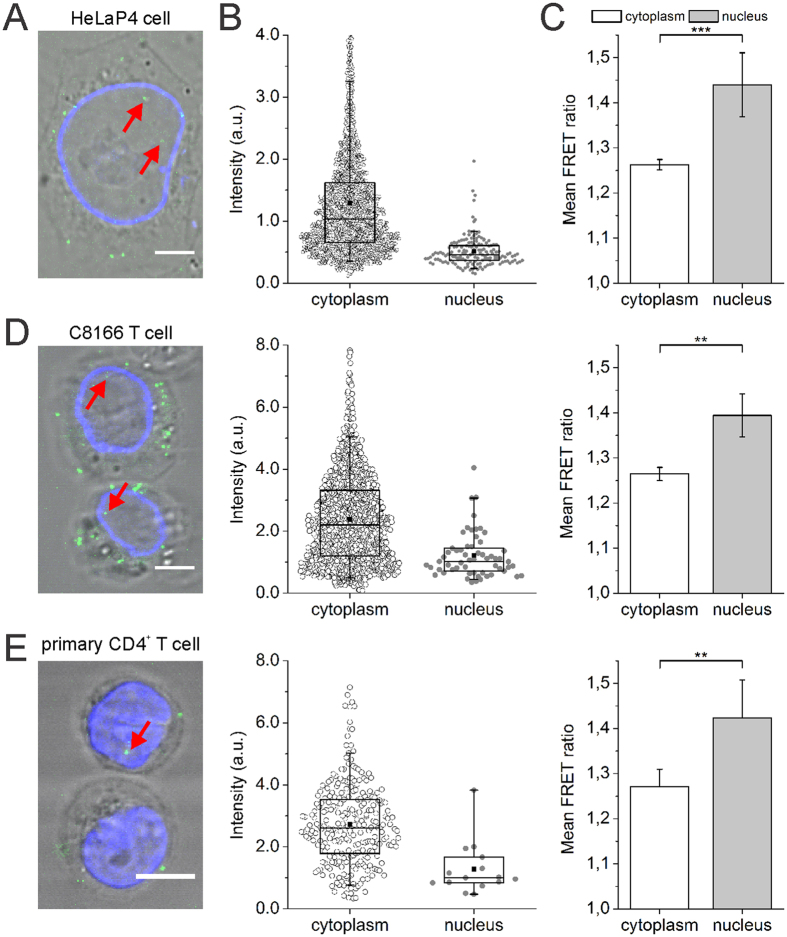
Decrease in fluorescence intensity and increase in FRET ratio in the nucleus of HeLaP4, C8166 T and primary CD4^+^ T cells. (**A**) Representative confocal image of a lamin immunostained (blue) HeLaP4 cell infected with VSV-G pseudotyped HIV_IN-eGFP_ for 6 h. Red arrows point towards nuclear IN-eGFP complexes. (**B**) Fluorescence intensity of HIV_IN-eGFP_ complexes in infected HeLaP4 cells in the cytoplasm (black circle) and in the nucleus (grey circle), obtained using the automated image analysis tool. Box-plot whiskers represent 5^th^ and 95^th^ percentile, the median value is represented by the line within the box, and the square box depicts the mean. (**C**) Mean FRET ratios of HIV_IN-mTFP1+IN-mVenus_ complexes in infected HeLaP4 cells in the cytoplasm (white) and in the nucleus (grey). (**D**) The left panel shows a representative confocal image of lamin immunostained (blue) C8166 T cells infected with HIV^Env^_IN-eGFP_, containing a wild-type HIV envelope, for 24 h. Red arrows indicate nuclear IN-eGFP complexes. Fluorescence intensity (middle) and mean FRET ratios (right) of HIV complexes in C8166 T cells infected with HIV^Env^_IN-eGFP_ and HIV^Env^_IN-mTFP1+IN-mVenus_, respectively. (**E**) The left panel shows a representative confocal image of DAPI stained (blue) CD4^+^ T cells infected with HIV^Env^_IN-eGFP_, containing a wild-type HIV envelope, for 24 h. Red arrows indicate nuclear IN-eGFP complexes. Fluorescence intensity (middle) and mean FRET ratios (right) are shown for HIV complexes in primary CD4^+^ T cells infected with HIV^Env^_IN-eGFP_ and HIV^Env^_IN-mTFP1+IN-mVenus_, respectively. Error bars represent standard error of mean (SEM); ***p-value < 0.001, **p-value < 0.01 obtained with an unpaired Two Sample t-test with unequal variance of the data. Scale bar represents 5 μm.

To quantify the amount of labeled IN inside a single viral complex, we calculated the fluorescence intensity of viral complexes in the different cellular compartments using our automated image analysis tool (Fig. 2A,B and Supplementary Table S2). As the fluorescence intensity appeared to be log-normally distributed, we used the geometric mean as a measure of intensity (Supplementary Fig. S4). Figure 2B reveals that cytoplasmic complexes have a two-fold higher intensity (1.035 ± 0.015 a.u.) compared to their nuclear counterparts (0.465 ± 0.016 a.u.), revealing that the latter contain less IN-eGFP molecules. To monitor the interaction between labeled IN subunits within viral complexes in the cell, we next quantified the FRET ratio. The FRET ratio of nuclear complexes (1.44 ± 0.07) was significantly higher than that in the cytoplasm (1.26 ± 0.01) (Fig. 2C and Supplementary Table S3), the latter value being similar with previous reported values for IN oligomers in the viral particles (1.27 ± 0.09^53^). We next sought to confirm these findings in cells more closely related to the physiological targets of HIV-1. Therefore we used a specific HIV enveloped virus (HIV^Env^) trans-complemented with the IN-FP fusion protein to infect a T cell line (C8166)^61^ (Fig. 2D and Supplementary Tables S2 and 3) as well as primary CD4^+^ T cells (Fig. 2E and Supplementary Tables S2 and 3). Analysis of these cells corroborated the reduced IN content of viral complexes in the nucleus and the accompanying increase in FRET ratio (Fig. 2D,E and Supplementary Fig. S5). Importantly, in separate experiments, we could exclude that the observed changes in fluorescence intensity and FRET ratio are due to different environmental conditions in the cytoplasm and nucleus (Supplementary Fig. S6). Furthermore, immunolabeled HA-tagged IN revealed a similar reduction in nuclear IN content compared to cytoplasmic PICs (Supplementary Fig. S7 and Supplementary Table S4), excluding the possibility that the reduced nuclear IN content is an artifact because of the bulky eGFP tag. Taken together, these results suggest that the viral complex undergoes considerable changes in its composition (and conformation) upon nuclear entry.

### Cellular cofactors affect IN oligomerization dynamics in the nucleus

**Figure 3:**
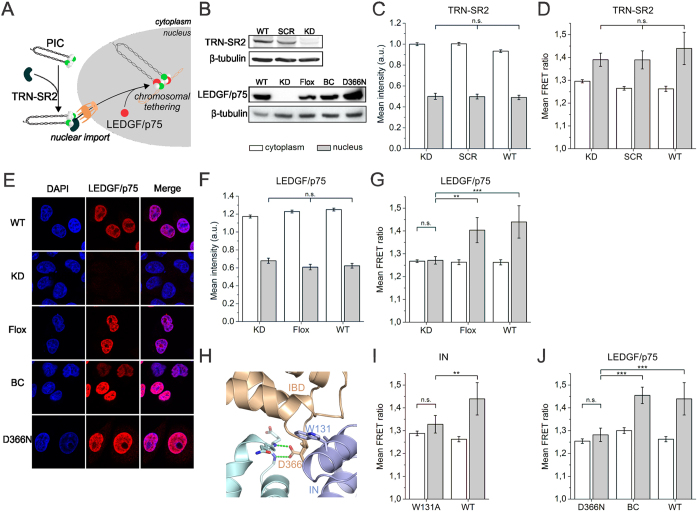
Cellular cofactor affects IN oligomerization dynamics in the nucleus. (**A**) The cellular cofactor TRN-SR2 has been implemented in HIV-1 nuclear import, while LEDGF/p75 tethers the PIC to the chromatin. (**B**) Western blot showing the depletion of TRN-SR2 and LEDGF/p75 respectively in TRN-SR2^KD^ and LEDGF/p75^KD^ cells. Other lanes represent LEDGF/p75 expression in the stable cell lines used in panel E. (**C,D**) Fluorescence intensity (**C**) and mean FRET ratios (**D**) in the cytoplasm (white) or nucleus (grey) in TRN-SR2^KD^, TRN-SR2^SCR^ and HeLaP4 WT cells infected with VSV-G pseudotyped HIV_IN-eGFP_ and HIV_IN-mTFP1+IN-mVenus_, respectively. (**E**) Representative confocal images of DAPI-stained (blue) and LEDGF/p75 immunostained (red) HeLaP4 cells. Row 1: HeLaP4 WT cells (WT), row 2: LEDGF/p75 depleted cells (LEDGF/p75^KD^), row 3: reversal of LEDGF/p75^KD^ by Cre specific excision of the LEDGF/p75 knockdown cassette reconstituting wild-type LEDGF/p75 (LEDGF/p75^Flox^), row 4 and 5: LEDGF/p75-depleted cells complemented with wild-type (LEDGF/p75^BC^) or mutant LEDGF/p75^D366N^. (**F,G**) Fluorescence intensity (**F**) and mean FRET ratios (**G**) in the cytoplasm (white) or nucleus (grey) in LEDGF/p75^KD^, LEDGF/p75^Flox^ and HeLaP4 WT cells infected with VSV-G pseudotyped HIV_IN-eGFP_ and HIV_IN-mTFP1+IN-mVenus_, respectively. (**H**) Crystal structure of the IN catalytic core dimer interface and the IBD of LEDGF/p75 (PDB 2B4J). The interface mutations W131A and D366N are indicated. (**I**) Mean FRET ratios in the cytoplasm (white) or nucleus (grey) in HeLaP4 cells infected with VSV-G pseudotyped HIV_IN-W131A-mTFP1+IN-W131A-mVenus_ compared to HIV_IN-mTFP1+IN-mVenus_. (**J**) Mean FRET ratios in the cytoplasm (white) or nucleus (grey) in LEDGF/p75^D366N^, LEDGF/p75^BC^ and LEDGF/p75^WT^ cells infected with HIV_IN-mTFP1+IN-mVenus_. SCR = scrambled; KD = knockdown; BC = back-complemented. Unless explicitly stated otherwise, the intensity and mean FRET ratios in the cytoplasm and nucleus were statistically different (*p*-value < 0.001). (***p-value < 0.001, **p-value < 0.01 and n.s. = not significant).

Several cellular cofactors are involved in the replication cycle of HIV-1. TRN-SR2, for example, is a known nuclear import factor of HIV-1 that interacts with IN (Fig. 3A)^11^,^12^,^20^. Hence, we infected HeLaP4 TRN-SR2 knockdown cells (TRN-SR2^KD^) and a scrambled control cell line (TRN-SR2^SCR^) (Fig. 3B) with HIV_IN-eGFP_ or HIV_IN-mTFP1+IN-mVenus_. As expected, previously observed defects in nuclear import were confirmed (Supplementary Table S2)^20^. In both the TRN-SR2^KD^ and TRN-SR2^SCR^ a loss of IN molecules in the nucleus was observed (Fig. 3C and Supplementary Table S2), coinciding with an increase in FRET ratio (Fig. 3D and Supplementary Table S3), similar to HeLaP4 WT cells. Thus, the change in the number of IN-eGFP molecules and the FRET ratio proved to be TRN-SR2 independent.

Next, we investigated the effect of LEGDGF/p75, a nuclear cofactor that stabilizes the tetrameric form of IN (Fig. 3A)^32^,^62^,^63^, by infecting HeLaP4 LEDGF/p75 knockdown cells (LEDGF/p75^KD^) with HIV_IN-eGFP_ or HIV_IN-mTFP1+IN-mVenus_. As a control for clonal selection artifacts, the LEDGF/p75 knockdown was reversed by Cre specific excision of the LEDGF/p75 knockdown cassette, reconstituting wild-type LEDGF/p75 expression (LEDGF/p75^Flox^) (Fig. 3B,E). A similar decrease in intensity of the nuclear HIV_IN-eGFP_ viral complexes was observed in both LEDGF/p75^KD^ and LEDGF/p75^Flox^ cells, implying that the presence of LEDGF/p75 does not modulate the number of IN-eGFP molecules in the nucleus (Fig. 3F and Supplementary Table S2). However, in the LEDGF/p75^KD^ cells no increase in nuclear FRET could be observed, contrary to the LEDGF/p75^Flox^ case, where the FRET ratio in the nucleus was similar to that of HeLaP4 WT cells, suggesting that LEDGF/p75 is required for the observed nuclear FRET phenotype (Fig. 3G and Supplementary Table S3).

To prove the specificity of the LEDGF/p75 requirement for this phenotype, we introduced the IN^W131A^ substitution, which is known to reduce the affinity of IN for LEDGF/p75 (Fig. 3H)^43^. Compared to HIV_IN-mTFP1+IN-mVenus_, HIV_IN-W131A-mTFP1+IN-W131A-mVenus_ was hampered in forming IN oligomers with increased FRET in the nucleus (Fig. 3I and Supplementary Table S3), which is in line with its effect on LEDGF/p75 binding. Complete disruption of the IN-LEDGF/p75 interaction is obtained by introducing the D366N substitution in LEDGF/p75^64^. Back-complementation cell lines expressing D366N (LEDGF/p75^D366N^) or WT LEDGF/p75 (LEDGF/p75^BC^) were generated and their expression was verified by western blot (Fig. 3B). Although LEDGF/p75 expression levels varied among individual cells (HeLaP4 WT, LEDGF/p75^Flox^, LEDGF/p75^BC^ and LEDGF/p75^D366N^) ubiquitous colocalization with chromatin was observed (Fig. 3E). As expected, the increase in FRET was rescued to WT levels in LEDGF/p75^BC^ cells (Fig. 3J and Supplementary Table S3). In contrast, in cells back-complemented with LEDGF/p75^D366N^, the nuclear FRET ratio was not restored but similar to that obtained with IN^W131A^ (Fig. 3I and Supplementary Table S3) and in the LEDGF/p75^KD^ cells (Fig. 3J and Supplementary Table S3). In conclusion, LEDGF/p75 but not TRN-SR2 orchestrates the interaction (and/or conformation) of IN subunits, yet does not define the quantity of IN molecules, in nuclear viral PICs.

### Nuclear IN reorganization in the PIC occurs in the absence of LEDGF/p75-mediated chromatin tethering

**Figure 4:**
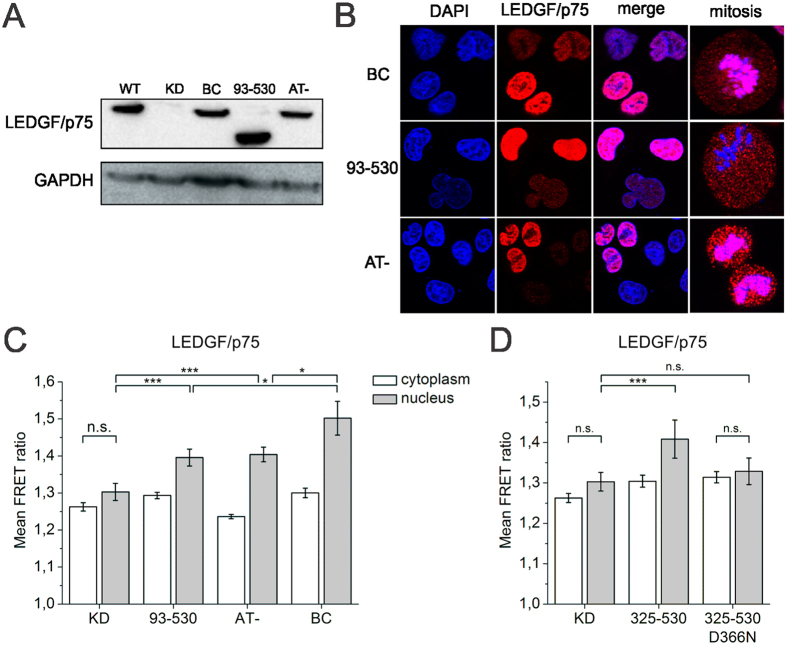
Nuclear IN reorganization in the PIC occurs in the absence of LEDGF/p75-mediated chromatin tethering. (**A**) Western blot showing LEDGF/p75 depletion in LEDGF/p75^KD^ cell lines and the complementation with the respective LEDGF/p75 truncations. (**B**) Representative confocal images of LEDGF/p75-depleted cells complemented with wild-type (LEDGF/p75^BC^) or mutant LEDGF/p75^93–530^ or LEDGF/p75^AT-^ during interphase and mitosis, stained for DAPI (blue) and immunostained for LEDGF/p75 (red). LEDGF/p75^BC^ but not LEDGF/p75^93–530^ binds mitotic chromatin. (**C**) Mean FRET ratios of VSV-G pseudotyped viral complexes (HIV_IN-mTFP1+IN-mVenus_) in the stable cell lines expressing the LEDGF/p75 constructs (LEDGF/p75^93–530^, LEDGF/p75^AT-^ and LEDGF/p75^BC^) compared to LEDGF/p75^KD^ cells. (**D**) Mean FRET ratios of VSV-G pseudotyped viral complexes (HIV_IN-mTFP1+IN-mVenus_) in LEDGF/p75^325–530^ and LEDGF/p75^325–530;D366N^ transfected LEDGF/p75^KD^ cells. White bars: cytoplasm; grey bars: nucleus; KD = knockdown; BC = back-complemented. Unless explicitly stated otherwise, the intensity and FRET ratios in the cytoplasm and nucleus were statistically different (*p*-value < 0.001). (***p-value < 0.001, *p-value < 0.05 and n.s. = not significant).

Whereas the LEDGF/p75 C-terminal part (IBD) interacts with HIV-1 IN, the N-terminal part of LEDGF/p75, consisting of a PWWP (Pro-Trp-Trp-Pro) domain, a nuclear localization signal (NLS), two AT-hooks and a supercoiled DNA recognition element ensures binding to chromatin and DNA^35^,^36^,^65^. To investigate which domains of LEDGF/p75 are involved in the nuclear FRET increase, we generated a series of LEDGF/p75 mutant constructs, specifically deleting or altering domains involved in chromatin binding (Supplementary Fig. S8) and reintroduced these to complement LEDGF/p75^KD^ cells. Expression of the LEDGF/p75 truncations was verified by western blot (Fig. 4A) and immunocytochemistry (Fig. 4B). As shown before, endogenous LEDGF/p75 was clearly excluded from nucleoli and localized to condensed chromatin during mitosis. Deletion of the PWWP domain (LEDGF/p75^93–530^) resulted in a more diffuse nuclear distribution and a loss of interaction with condensed chromatin (Fig. 4B)^66^. Complementation with the AT-hook mutant LEDGF/p75^AT-^ (R183D, K192D and R196D)^67^ resulted in a similar localization as LEDGF/p75^BC^ (Fig. 4B). In LEDGF/p75^93–530^ and LEDGF/p75^AT-^ cells infected with HIV_IN-mTFP1+IN-mVenus_ an increased FRET signal was observed for the nuclear complexes similar to LEDGF/p75^BC^ cells (Fig. 4C and Supplementary Table S3), showing that chromatin tethering is not absolutely required to increase the FRET ratio in the nucleus.

Finally, we generated a construct encoding the C-terminal moiety of LEDGF/p75 (residues 325–530, including the IBD) fused to the C-terminus of a non-fluorescent eGFP^Y66L^ to increase protein stability (LEDGF/p75^325–530^)^68^. As a negative control we introduced the D366N mutation in the IBD to interfere with the interaction with HIV-1 IN (LEDGF/p75^325–530;D366N^). Both constructs were transiently expressed in LEDGF/p75^KD^ cells and these cells were subsequently infected with HIV_IN-mTFP1+IN-mVenus_ particles. Noteworthy, an increased FRET signal in the nucleus of the LEDGF/p75^325–530^ expressing cells was observed, which was absent in the IN interaction-deficient LEDGF/p75^325–530^ expressing cells (LEDGF/p75^325–530;D366N^) (Fig. 4D and Supplementary Table S3). In conclusion, binding of the C-terminal part of LEDGF/p75 to IN is sufficient to increase the FRET signal in the nucleus, confirming its role in the nuclear dynamics of IN oligomers within the HIV PIC.

### The nuclear pore acts as a molecular filter for HIV-1 nuclear import

**Figure 5:**
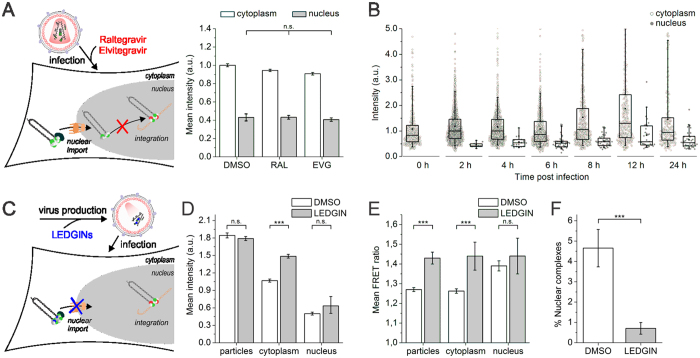
The nuclear pore acts as a molecular filter, impeding the nuclear import of LEDGIN pre-treated viral particles. (**A**) Fluorescence intensity of VSV-G pseudotyped HIV complexes (HIV_IN-eGFP_) in the cytoplasm (white) or nucleus (grey) of cells treated with DMSO, RAL (0.6 μM) or EVG (0.2 μM) during infection. (**B**) Fluorescence intensity of single IN-eGFP complexes in the cytoplasm (white circles) and nucleus (grey circles) at different time points post infection (0–24h). (**C**) Nuclear import of particles produced in the presence of DMSO or LEDGINs (1.2 μM, CX14442) is hampered. (**D**) Fluorescence intensity of viral particles and viral complexes localized in the cytoplasm or nucleus of HeLaP4 WT cells infected with VSV-G pseudotyped HIV_IN-eGFP_ particles produced in the presence of DMSO (white) or LEDGINs (1.2 μM, CX14442) (grey). (**E**) FRET ratios of viral particles and viral complexes localized in the cytoplasm or nucleus in HeLaP4 WT cells infected with VSV-G pseudotyped HIV_IN-mTFP1+IN-mVenus_ particles produced in the presence of DMSO (white) or LEDGINs (1.2 μM, CX14442) (grey). (**F**) Percentage nuclear complexes (HIV_IN-eGFP_) in HeLaP4 cells infected with VSV-G pseudotyped particles produced in the presence of DMSO or LEDGINs (1.2 μM, CX14442). Unless explicitly stated otherwise, the intensity and FRET ratios in the cytoplasm and nucleus were statistically different (*p*-value < 0.001). (***p-value < 0.001 and n.s. = not significant).

While previous experiments point to LEDGF/p75 as the main determinant of the increased nuclear FRET ratio, the difference in the number of IN molecules per complex between cytoplasm and nucleus was LEDGF/p75 independent. Analysis of the spread in fluorescence intensity of single viral complexes revealed that the number of IN-eGFP molecules per complex varies considerably in the cytoplasm (1.035 ± 0.972 a.u. (geometric mean ± SD)) (Fig. 2B). This broad range in IN content possibly reflects a heterogeneous population consisting of functional and non-functional viral particles, with defects in maturation, uncoating or reverse transcription, potentially influenced by a wide array of host defense mechanisms^69^. In stark contrast, the spread in intensity was much less pronounced among nuclear complexes (0.465 ± 0.239 a.u. (geometric mean ± SD)) (Fig. 2B). In theory these nuclear IN complexes may include functional intasomes, inactive IN aggregates or post-integration IN complexes. Since the same low intensity nuclear IN complexes were observed after inhibition of integration with INSTIs (RAL or EVG) (Fig. 5A, Supplementary Fig. S9 and Supplementary Table S5), we conclude that integration is not required to reduce the number of IN in nuclear PICs. Alternatively, the nuclear complexes may contain less IN due to gradual intasome degradation over time. This would imply the existence of complexes with varying intensities in the nucleus, starting with high intensity complexes early post infection and the accumulation of low intensity particles over time. To investigate this possibility we performed a time-course, after a synchronized infection by incubating HIV_IN-eGFP_ for two hours at 16 °C followed by a temperature shift to 37 °C which enables viral fusion^70^. HIV_IN-eGFP_ complexes reached the cytoplasm already after two hours of incubation at 16 °C (zero hour time point) indicating endocytosis of VSV-G-pseudotyped virus or virus-like particles at 16 °C, resulting in incomplete synchronization. Nevertheless, at all time points tested from 2 to 24 h post infection, nuclear IN complexes displayed the same reduced intensity and spread (Fig. 5B and Supplementary Table S6). Together, these observations suggest that all HIV complexes that enter the nucleus already have a reduced intensity. We conclude from this analysis that the nuclear pore may act as a molecular filter, granting passage into the nucleus only to those complexes which have a reduced number of IN molecules.

To corroborate this hypothesis we produced viral particles in the presence of LEDGINs, which are known to enhance IN multimerization in newly formed particles (Fig. 5C). Indeed, production of HIV_IN-mTFP1+IN-mVenus_ in the presence of LEDGINs resulted in a significantly higher FRET ratio (1.43 ± 0.03) (Fig. 5E)^49^,^53^. Remarkably, also the intensity of cytoplasmic IN complexes of pretreated virus was higher than in the untreated controls (Fig. 5D and Supplementary Table S7), evidencing the higher IN content of these complexes. Since DMSO and LEDGIN-treated HIV_IN-eGFP_ particles had the same initial fluorescence intensity, equal amounts of IN-eGFP were present in the virus, and Vpr-transincorporation was unaffected (Fig. 5D and Supplementary Table S7). When infecting HeLaP4 WT cells with LEDGIN-treated HIV_IN-mTFP1+IN-mVenus_ viral particles, an invariable FRET ratio was observed for the HIV complexes in the cytoplasm (1.39 ± 0.03) and the nucleus (1.44 ± 0.09) (Fig. 5E and Supplementary Table S7) indicating that the enhanced multimerization state of IN inside the viral particle is retained throughout the early replication steps. Together with the intensity data, we therefore propose that LEDGIN-induced IN multimerization during virus assembly results in an overly stable association of IN molecules. Hence, the nuclear import defect reported for LEDGIN pre-treated virus (Fig. 5F)^49^ may be directly related to the presence of this larger, static IN complex (LEDGIN-induced multimerization), which could be excluded from the nucleus. Taken together, these results suggest that the nuclear pore acts as a selective molecular filter for HIV nuclear import.

### LEDGINs modulate nuclear but not cytoplasmic IN oligomers

**Figure 6:**
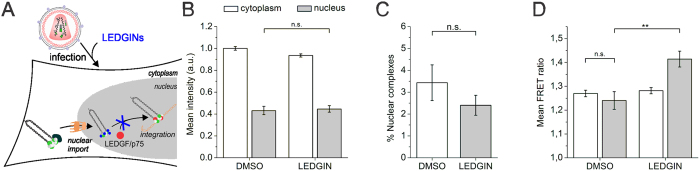
LEDGINs alter the IN oligomer in the nucleus. (**A**) LEDGINs inhibit the interaction between LEDGF/p75 and IN by binding to the IN oligomer. (**B**) Fluorescence intensity of VSV-G pseudotyped HIV_IN-eGFP_ complexes in the cytoplasm (white) or nucleus (grey) of HeLaP4 WT cells treated with DMSO or LEDGINs (45 μM, CX14442) during infection. (**C**) Percentage nuclear IN-eGFP complexes in HeLaP4 WT cells treated with DMSO or LEDGINs (45 μM, CX14442) during infection. (**D**) Mean FRET ratios of VSV-G pseudotyped HIV_IN-mTFP1+IN-mVenus_ complexes in the cytoplasm (white) or nucleus (grey) of LEDGF/p75^KD^ cells treated with DMSO or LEDGINs (45 μM, CX14442) during infection. Unless explicitly stated otherwise, the intensity and FRET ratios in the cytoplasm and nucleus were statistically different (*p*-value < 0.001). (**p-value < 0.01 and n.s. = not significant).

Since LEDGINs are known to inhibit integration, we also investigated their effect on early stage replication. Upon addition of LEDGINs during infection (Fig. 6A) the fluorescence intensity of cytoplasmic HIV_IN-eGFP_ complexes remained equal to that in the DMSO treated controls. Likewise the decrease in intensity upon entry in the nucleus was unaffected (Fig. 6B and Supplementary Table S8). Thus, LEDGINs do not affect the number of IN-eGFP molecules in the cytoplasm nor the loss of IN-eGFP upon nuclear entry. Nuclear import was not affected by LEDGINs when added during infection (Fig. 6C and Supplementary Table S5).

Since we have shown in this study that LEDGF/p75 increases the nuclear FRET ratio, we performed FRET experiments in LEDGF/p75^KD^ cells in order to discriminate between possibly overlapping effects of LEDGINs and of LEDGF/p75. Interestingly, LEDGIN treatment during infection increased the FRET ratio in the nucleus, while that of the cytoplasmic HIV_IN-mTFP1+IN-mVenus_ complexes was similar to that in the DMSO treated controls (Fig. 6D and Supplementary Table S8). Hence, only after entering the nucleus LEDGINs were able to modulate the IN conformation. In conclusion, LEDGINs modulate the IN oligomer but only after nuclear entry, pinpointing a second IN multimerization state-sensitive phase in the HIV replication cycle aside from viral particle assembly.

## Discussion

To date, molecular details of nuclear entry of the HIV PIC remain obscure, therefore necessitating an experimental approach that allows single-virus analysis to address viral heterogeneity and avoid population averaging. Here we provide evidence for an HIV-1 nuclear entry/integration pathway orchestrated by the number and oligomeric state of integrase in the PIC. Since HIV-1 IN plays a major role throughout early replication, we probed HIV particles containing fluorescently labeled IN using sensitive single-virus microscopy techniques. By determining the fluorescence intensity of single viral particles (Fig. 1A; Middle), which is directly proportional to the number of labeled IN molecules, we assessed alterations in IN content during nuclear entry of the PIC (Figs 2 and 5). Additionally, we measured FRET via acceptor photobleaching (Fig. 1A; Bottom)^53^ to quantitatively study the interaction between different IN subunits as well as the role of cellular cofactors in modulating IN oligomerization upon nuclear entry (Figs 2, 3 and 4). Finally, we provide a mechanism to explain why viral particles assembled in the presence of LEDGINs fail to enter the nucleus (Figs 5 and 6).

### Single virus microscopy, a novel approach to study nuclear import and inhibitors

By using an HIV packaging construct that encodes WT IN instead of the catalytically defective IN^D64E ^ mutant^20^,^53^,^54^,^55^, fluorescently labeled viral particles were generated with WT infectivity (Fig. 1B). Addition of the INSTIs, RAL or EVG, increased the number of nuclear IN-FP complexes (Fig. 1D). Since INSTIs only recognize IN assembled on processed vDNA into a specific intasome complex^29^,^71^, at least a subset of the detected nuclear IN-FP complexes must contain a functional intasome. In contrast, upon treatment with the CA-binding inhibitor PF74, hardly any IN-eGFP complexes were detected in the nucleus, which is in agreement with the reported drop in 2-LTR circle formation (Fig. 1C,D)^6^,^7^,^56^,^57^. Hence, single virus microscopy can be applied to study inhibitors of early steps of the HIV-1 replication cycle.

Detection of HIV complexes and quantification of their total fluorescence intensity using our automated analysis routine (Supplementary Fig. S2) offers an alternative for methods which count a limited number of HIV complexes by manually selecting a region of interest (ROI) and sum or average pixel intensities^6^,^55^,^70^. In our routine ROIs are automatically determined using an intensity threshold, which results in a higher precision and the unbiased processing of thousands of complexes within a few hours. Furthermore, the fluorescence intensity is quantified by fitting the PSF of each detected complex with a 2D Gaussian function, which automatically generates and subtracts an offset corresponding to the local background and is less affected by noise from the detector and excitation light fluctuations.

### Upon nuclear entry, HIV IN complexes are modulated

Nuclear entry in HeLaP4 cells, a T cell line and primary T cells was associated with a modulation in both the composition of the IN multimer and its interactions (Fig. 2). The chromatin tethering factor LEDGF/p75 but not the nuclear import factor TRN-SR2 proved to be the host factor responsible for increasing the IN FRET signal in the nucleus of HeLaP4 cells (Fig. 3). While a direct and Ran-dependent interaction between IN and TRN-SR2 was verified *in vitro*^20^,^72^, no correlation between formation of IN tetramers and TRN-SR2 binding has been observed^73^. The FRET data agree with the hypothesis that TRN-SR2 has no direct influence on IN oligomerization^73^. In contrast, LEDGF/p75 has originally been identified in a cellular complex with multimeric IN^32^ and the protein is known to modulate the dynamic structure of HIV-1 IN by stabilizing subunit-subunit interactions^63^. Although we cannot formally exclude an indirect effect of LEDGF/p75, inhibition of the interaction by a single interface substitution in IN-FP (IN^W131A^)^43^ or LEDGF/p75 (LEDGF/p75^D366N^) prevented the formation of a higher FRET oligomer (Fig. 3I,J). The modulation of the IN complex architecture by LEDGF/p75 occurs upon nuclear entry, is mediated through the C-terminus of LEDGF/p75 and is at least in part independent of chromatin tethering (Fig. 4C,D). Interestingly, whereas overexpression of the C-terminus of LEDGF/p75 in the nucleus of LEDGF/p75-depleted cells rescued the increase in FRET signal, cytoplasmic expression could not (Fig. 4D). Although De Rijck *et al.* showed that both transiently overexpressed mRFP-IN and eGFP-LEDGF/p75^325–530^ can interact in the cytoplasm^68^, our experiments indicate that IN in the context of a PIC is inaccessible for LEDGF/p75 prior to nuclear entry. This analysis is in line with mounting evidence for a capsid cone that shields HIV proteins and DNA from the cellular environment during trafficking to the nuclear membrane^7^. The fact that LEDGF/p75 only engages IN upon nuclear entry might suggest that capsid uncoating only takes place at the nuclear pore as suggested by Arhel *et al.*^3^,^74^. Whether the observed reduction in IN upon nuclear entry is also required for LEDGF/p75 interaction still needs to be investigated.

Apart from LEDGF/p75-induced IN multimerization, a second important alteration in the IN multimers was observed. Nuclear HIV PICs contain two-fold less IN-eGFP molecules than their cytoplasmic counterparts (Fig. 2B) and the nuclear population is considerably more homogeneous than the cytoplasmic with regard to IN content (Figs 2B and 5B). Using superresolution microscopy Lelek *et al.* showed that cytoplasmic PICs are generally larger in size than nuclear PICs^75^. Here we propose that IN content determines nuclear import. Since the NPC only allows access of macromolecules up to ~39 nm^76^, and since we also observed this IN ‘shedding’ in a T cell line and in primary CD4^+^ T cells infected with HIV-1 enveloped virus (Fig. 2D,E), we propose that the function as a size filter of the IN complex is a general feature for productive HIV nuclear import.

DNA viruses and retroviruses evolved different strategies to overcome this filter^5^,^77^. All seem to use Nup358 as docking platform. Whereas hepatitis B virus (~27 nm)^78^ enters the nuclear pore to uncoat and releases viral DNA upon arrival in the nuclear basket, the much larger herpes simplex virus (100 nm)^79^ uncoats on the cytoplasmic side of the nuclear pore. The HIV capsid cone is 100–150 nm long and 40–60 nm wide^80^, which is in between that of hepatitis B virus and the herpes simplex virus, requiring at least partial uncoating of the PIC for nuclear access^8^,^9^.

### The mechanism of action of LEDGINs provides evidence for the nuclear pore filter

LEDGINs, small molecules recognizing the LEDGF/p75 binding pocket on HIV-1 IN, interfere with various steps of HIV-1 replication^37^,^44^. By abrogating the IN-LEDGF/p75 interaction and promoting unproductive higher order IN oligomerization they inhibit integration. Several groups confirmed that LEDGINs enhance IN multimerization within newly formed viral particles leading to maturation defects^49^,^51^,^52^,^53^. Here we show that the higher IN FRET signal obtained after LEDGIN treatment during virus production is retained during infection (Fig. 5E), arguing for very stable IN oligomeric states and a seemingly irreversible process. More importantly, LEDGIN-enhanced IN multimerization during assembly prevents the loss of IN molecules that is required for nuclear import in HeLaP4 cells because of the size limitation as shown here (Fig. 5D,E). Remarkably, the FRET ratio in the cytoplasm of cells infected with LEDGIN-treated viral particles is comparable with the LEDGF/p75-induced value observed in the nucleus with untreated virus (1.44 ± 0.07) (Fig. 5E). Although we cannot conclude that the underlying mechanism is exactly the same, LEDGINs may mimic the IN alteration obtained by interaction with nuclear LEDGF/p75 prematurely in the viral particles. Importantly, addition of LEDGINs during infection only increased the FRET signal in the nucleus but not in the cytoplasm (Fig. 6D). Since inaccessibility of the PIC to a small molecule is unlikely, the IN oligomeric and/or conformational state in the cytoplasm seems to preclude modulation by LEDGINs. We cannot exclude that the observed IN ‘shedding’ upon nuclear entry is required for LEDGIN interaction. Alternatively, minute amounts of LEDGF/p75 or its C-terminus in the PIC^81^ may compete with LEDGIN binding in the cytoplasm. Although LEDGINs increase the FRET signal of IN to the same extent as LEDGF/p75, these ratios may correspond to entirely different complexes as suggested before^82^. In any case, the experiments with LEDGINs and LEDGF/p75 consistently indicate extensive changes in the IN multimer conformation and/or composition upon nuclear entry.

**Figure 7:**
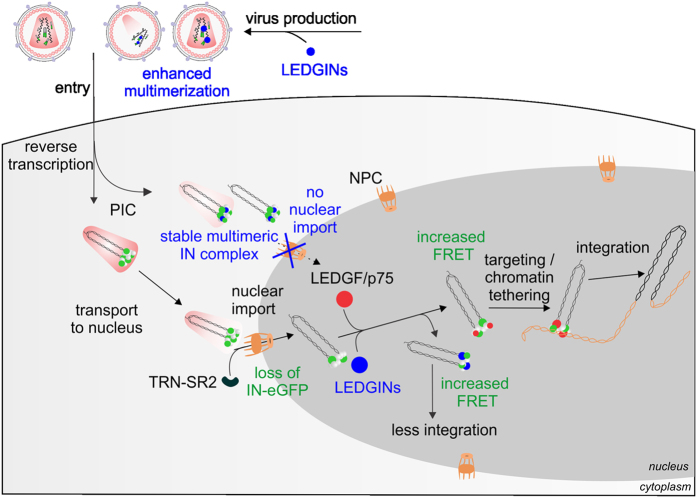
Model for nuclear entry of IN labeled PIC. The early stages of the HIV replication cycle in an infected cell are shown. After infection of cells, WT IN (gray circle) and labeled IN (green circle) associated with reverse transcribed vDNA are imported into the nucleus assisted by TRN-SR2 as part of the pre-integration complex (PIC). Nuclear entry of the PIC requires loss of IN-eGFP and is inhibited when stable multimeric IN complexes are present. Stable multimeric complexes are formed when LEDGINs (blue circles) are present during the production of new virus. LEDGINs can bind to the polyprotein precursor containing IN or to the mature IN. When bound, LEDGINs enhance IN multimerization forming stable multimeric complexes and a block in nuclear import. Upon nuclear entry of functional complexes, LEDGF/p75 (red circle) binds to the IN complex, resulting in an oligomer with a higher FRET ratio, activated for efficient integration. When LEDGINs (blue circle) are present during PIC nuclear import, they compete with LEDGF/p75 for binding to IN. LEDGINs inhibit integration by preventing IN-LEDGF/p75 complex formation and enhancing IN oligomerization.

Based on our results we propose the molecular mechanism of LEDGINs shown in Fig. 7. Upon nuclear entry through the NPC the IN complex is modulated. First, a large part of the viral proteins, including labeled IN molecules, which are not tightly associated with the vDNA or other PIC components, are lost. Our data do not contradict the growing evidence that capsid uncoating takes place at the nuclear membrane^74^. Still, from our data we cannot conclude whether complexes which already have a reduced number of IN-eGFP molecules are the only ones that can enter or whether active IN uncoating takes place at the NPC. Subsequently, the vDNA associated with at least four IN molecules (but probably more) is imported in the nucleus.

Second, upon arrival in the nucleus, the IN complex is modulated by LEDGF/p75. We consider a scenario according to which IN first assembles on the vDNA ends^29^, after which the IN tetramer-vDNA ends complex engages LEDGF/p75^83^. Finally, this nucleoprotein complex is tethered by LEDGF/p75 to active transcription units and primed for successful integration. By enhancing IN multimerization prematurely, LEDGINs interfere with virus assembly, reverse transcription, IN uncoating and thus nuclear import, IN oligomerization dynamics upon nuclear entry and chromatin tethering. This multifaceted mechanism of action explains their antiviral potency. Collectively, using the power of single-virus analysis to study the fate of HIV IN during viral replication we show for the first time the requirement for a PIC IN ‘uncoating step’ during nuclear entry as well as a role of LEDGF/p75 in directing the oligomerization or conformational dynamics of IN in the nuclear PIC.

## Materials and Methods

### Virus production for fluorescence intensity and FRET measurements

Vesicular stomatitis virus glycoprotein (VSV-G)-pseudotyped HIV-1-derived viral particles containing fluorescently labeled IN (HIV_IN-eGFP_ and HIV_IN-mTFP1+IN-mVenus_) were generated by Vpr-mediated trans-incorporation^53^,^54^. 6.5 × 10^6^ HEK293T producer cells were seeded per 10 cm petri dish in DMEM supplemented with 2% FBS. At a density of 90%, cells were transfected using branched polyethylenimine (bPEI, 10 μM, Sigma-Aldrich) with 5 μg pVSV-G, 15 μg pNL4–3.Luc.R^−^.E^−^ and a specific amount of Vpr-IN-FP-encoding plasmid. HIV_IN-eGFP_ was produced with 15 μg of Vpr-IN-eGFP plasmid, while HIV_IN-mTFP1+IN-mVenus_ was produced with 2 μg of each Vpr plasmid per dish (Vpr-IN-mTFP1 and Vpr-IN-mVenus). Six hours post-transfection, the medium was replaced with pre-warmed OptiMEM (Life Technologies) supplemented with 50 μg/mL gentamicin (Invivogen). Supernatant was collected 48 h post-transfection, filtered through a 0.45 μm filter (Sartorius), and concentrated by ultracentrifugation on a 60% (w/v) iodixanol cushion on room temperature (131,500 g, 90 min, SW28 rotor, Beckman Coulter, Ireland). Next, the iodixanol was removed by ultrafiltration (Vivaspin, MWCO 50K, Merck, Overijse, Belgium). For the production of viruses in the presence of LEDGINs or DMSO, the transfection medium was replaced with fresh OptiMEM supplemented with CX14442 (1.2 μM, 20 × IC_50,_ synthesized by the KU Leuven Centre for Drug Design and Development (CD3)) or an equal volume of DMSO. HIV-enveloped viral particles containing fluorescently labeled IN (HIV^Env^_IN-eGFP_ and HIV^Env^_IN-mTFP1+IN-mVenus_) were produced in a similar way, with the following changes: cells were transfected with 20 μg pNL4-3 and 15 μg of Vpr-IN-eGFP plasmid, or 2 μg Vpr-IN-mTFP1 and Vpr-IN-mVenus plasmid, supernatant was collected 48 and 72 h post transfection and the virus was concentrated by ultrafiltration (Vivaspin, MWCO 50K) without ultracentrifugation.

### Infection of HeLaP4 cells

3 × 10^4^ HeLaP4 cells were seeded per well in poly-D-lysine (0.1 mg/ml, Sigma) coated 8-well chambered cover glasses (Nunc Lab-Tek Chambered Coverglasses, 155411, Thermo Scientific) and infected the next day with an amount (1–4 μg of p24 antigen) of HIV_IN-eGFP_ or HIV_IN-mTFP1+IN-mVenus_. Different viral preps (e.g. production with DMSO or LEDGINs) were normalised on p24 antigen content in order to infect with the same amount of virus. Six hours post-infection, cells were briefly incubated with trypsin (0.25% (w/v), 30 s, Life Technologies), washed with PBS and fixed with 4% (v/v) paraformaldehyde. See Supplementary methods for immunocytochemistry. When inhibitors PF-03450074 (PF74) (10 μM, SML0835, Sigma), raltegravir (0.6 μM, 100 × IC_50_, NIH AIDS Reagent Program, Division of AIDS, NIH (Cat #11680) from Merck & Company, Inc), elvitegravir (0.2 μM, 100 × IC_50_), CX14442 (30–45 μM) or DMSO were used during infection, cells were first pre-incubated for 2 h with the respective compound, after which the virus was added in the presence of the inhibitor for 6 h. See Supplementary methods for infection of C8166 T cells and CD4^+^ T cells. For time course measurements infection was synchronised. Therefore, pre-cooled HeLaP4 cells (15 min at 4 °C) were infected for 2 h at 16 °C (zero time point) followed by a temperature shift to 37 °C to allow viral entry^70^. After 2 h of incubation at 37 °C (2 h time point), cells were washed with pre-warmed OptiMEM, and further incubated to reach a total infection time of 24 h.

### Confocal microscopy and data analysis for nuclear import and intensity measurements

Imaging of cells and viral particles was performed using a laser scanning microscope (Fluoview FV1000, Olympus, Tokyo, Japan). See Supplementary methods for specifications of the microscope and image acquisition.

The automatic detection, localization and intensity calculation of each IN-eGFP complex was performed using a homemade MatLab routine (The MathWorks, Inc.) (See also Supplementary Fig. S2). In brief, after image processing, particles were automatically detected using an intensity threshold. This threshold is calculated using the triangle algorithm^84^ with addition of the double of the mean image intensity of each *z*-slice. Each fluorescent spot that consisted of less than 7 pixels above the threshold was excluded. The remaining spots were assigned to be part of a single IN-eGFP complex if they were observed in at least 3 consecutive *z*-slices, with the complex at a maximum distance of 3 pixels (309 nm). Large aggregates were removed manually. Subsequently, the nuclear lamin was determined using intensity thresholding (triangle algorithm with addition of the mean image intensity), and then used for automatic assignment of complexes to cytoplasm or nucleus. The percentage of nuclear complexes per cell was calculated and the mean over all cells represents the final percentage of nuclear IN-eGFP complexes. Since the fluorescence signal of each complex is diffraction limited, the point spread function of each spot was fitted with a 2D Gaussian using the least-mean-square method to calculate the integrated intensity of each IN-eGFP complex. The integrated intensity of this function was calculated in each *z*-slice. Since the *z*-slice in which the complex is in focus is expected to have the highest intensity, this intensity value was selected for further use. Complexes for which the fitted 2D Gaussian had widths (σ_x_ and σ_y_) larger than 2 pixels were excluded since these are assumed to be aggregates. Typically, data were collected from at least 30 different cells, which correspond to at least 1000 detected complexes per experiment. Each experiment was performed at least twice and data from a representative experiment are shown. The mean percentage of nuclear complexes is presented in a bar diagram, with the standard error of mean (SEM) as error bars. P-values are obtained from a Mann-Whitney test. Intensity data are presented as individual data points overlaid with a box-plot, whiskers representing the 5th and 95th percentiles. The median value is represented by the line within the box and the square box depicts the mean. Bar diagrams represent the geometric mean of the intensity data, error bars are the back-transformed standard error of the mean (See also Supplementary Fig. S4). P-values for the intensity data are obtained from a Two-sample t-test with unequal variance of the log-normal distributed data with *p*-value < 0.001 (***), *p* < 0.01 (**), *p* < 0.05 (*) and n.s. = not significant as the criterion of significance.

### Quasi-TIRF microscopy and data analysis for FRET measurements

FRET measurements were performed on an inverted microscope (Olympus IX-83, Olympus NV). See Supplementary methods for specifications of the microscope and image acquisition. Single viral complexes (HIV_IN-mTFP1+IN-mVenus_) were localized by dual-color 2D Gaussian localization of the sub diffraction viral complexes (100–150 nm). Subsequently, the single-particle integrated fluorescence intensity per virus of the FRET donor before (*F*_D,pre_) and after (*F*_D,post_) photobleaching and of the FRET acceptor was extracted. Single-molecule localization/fitting was performed with Localizer^85^. FRET was finally quantified by calculating the FRET ratio^86^:
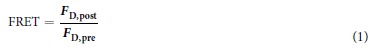
If FRET occurred between donor and acceptor, an increase of the donor fluorescence is observed after photobleaching of the acceptor. Typically, data were collected from ~50–100 different cells, corresponding to an average of ~200–2000 viral complexes per measurement. From all the obtained FRET ratios, the average was taken and the error bars on the bar graphs represent the standard error of the mean (SEM). Statistical analyses were performed with a Two-sample t-tests with unequal variance with the following *p*-value cutoffs: *p* < 0.001 (***), *p* < 0.01 (**), *p* < 0.05 (*) and n.s. = not significant.

### Ethics statement

The human peripheral blood mononuclear cells were isolated from anonymous healthy blood donors. Buffy coats were obtained from the Red Cross Blood transfusion Center (Mechelen, Belgium) according to approved bioethical guidelines (S57175-IRB00002047) of the ethical committee of our institute, with informed consent of all donors. All methods were carried out in accordance with the guidelines and regulations of KU Leuven. All experimental protocols were approved by the HSE department of KU Leuven.

## Additional Information

**How to cite this article**: Borrenberghs, D. *et al.* Dynamic Oligomerization of Integrase Orchestrates HIV Nuclear Entry. *Sci. Rep.* **6**, 36485; doi: 10.1038/srep36485 (2016).

**Publisher’s note:** Springer Nature remains neutral with regard to jurisdictional claims in published maps and institutional affiliations.

## Supplementary Material

Supplementary Information
