# Supplementary material for: Dynamic Oligomerization of Integrase Orchestrates HIV Nuclear Entry
Source: Sci Rep. 2016 Nov 10;6:36485. doi: 10.1038/srep36485 (PMC5103197; doi:10.1038/srep36485)
Supplement: Supplementary Information [file srep36485-s1.pdf]

Dynamic Oligomerization of Integrase Orchestrates HIV Nuclear Entry

Doortje Borrenberghs [1,2,]*, Lieve Dirix [1,2,]*, Flore De Wit [1], Susana Rocha [2], Jolien Blokken [1], Stéphanie De Houwer [1], Rik Gijsbers [3], Frauke Christ [1], Johan Hofkens [2], Jelle Hendrix [2$] and Zeger Debyser [1$]

[1] Laboratory for Molecular Virology and Gene Therapy, Department of Pharmaceutical and Pharmacological Sciences, KU Leuven, Leuven, 3000, Belgium

[2] Laboratory for Photochemistry and Spectroscopy, Molecular Imaging and Photonics, Department of Chemistry, KU Leuven, Heverlee, 3001, Belgium

[3] Laboratory for Viral Vector Technology & Gene Therapy, Department of Pharmaceutical and Pharmacological Sciences, KU Leuven, Leuven, 3000, Belgium

* These authors contributed equally to this work

[$] zeger.debyser@kuleuven.be and jelle_hendrix@hotmail.com

Jelle Hendrix's present address is Faculty of Medicine and Life Sciences and Biomedical Research Institute, Hasselt University, Diepenbeek, 3590, Belgium

Supplementary Information

SUPPLEMENTARY TABLES

Table S1. Intensity of TetraSpeck Microspheres and $HIV_{IN\text{-}eGFP}$ with Reduced Amounts of Vpr-IN-eGFP plasmid. Related to Supplementary Figure S3.

	**TetraSpeckTM Microspheres[(a)]**	
***z*-position (µm)[(b)]**	**Intensity[(c)]**	**N[(d)]**
0.75	1.076 ± 0.309	31
2.25	1.091 ± 0.571	32
3.75	0.970 ± 0.404	28
5.25	0.942 ± 0.225	28
6.75	0.923 ± 0.262	34
8.25	1.001 ± 0.248	24
9.75	0.949 ± 0.182	25
11.25	0.987 ± 0.349	24
12.75	1.067 ± 0.473	25
14.25	0.947 ± 0.200	35
	**$HIV_{IN\text{-}eGFP}$[(e)]**	
**Vpr-IN-eGFP (µg)[(e)]**	**Intensity[(f)]**	**N[(g)]**
5.0	1.685 ± 0.075	117
7.5	1.857 ± 0.088	133
10	2.174 ± 0.092	133
15	2.248 ± 0.086	189

[(a)] 100 nm TetraSpeckTM Microspheres were embedded in agarose.

[(b)] Microspheres within a 1.5 µm slice were binned, the bin centre is shown.

[(c)] Each individual microsphere was localized and fitted with a 2D Gaussian curve, and the integrated intensity was calculated in each *z*-slice. The mean of the intensity data is shown; ± represents the standard deviation within each bin.

[(d)] N is the number of single microspheres within each bin used to calculate the mean of the intensity data

[(e)] $HIV_{IN\text{-}eGFP}$ was produced with the respective amount of Vpr-IN-eGFP plasmid per petri dish.

[(f)] Each individual viral particle was localized and fitted with a 2D Gaussian curve, and the integrated intensity was calculated in each *z*-slice. The mean of the intensity data is shown, ± represents the standard error of the mean (SEM).

[(g)] N is the number of single viral particles used to calculate the mean of the intensity data.

Table S2. Intensity of $HIV_{IN\text{-}eGFP}$ Viral Complexes. Related to Figure 2 and 3.

**$HIV_{IN\text{-}eGFP}$**	**Cytoplasm**		**Nucleus**	
**Cell type[b]**	**Intensity[c]**	**N[d]**	**Intensity[c]**	**N[d]**
HeLaP4[a]	1.035 ± 0.015	2193	0.465 ± 0.016	153
C8166 T cell line[e]	2.200 ± 0.029	1246	1.046± 0.079	58
$CD4^+$ T cell[e]	2.613 ± 0.057	261	1.002 ± 0.068	15
HeLaP4 TRN-$SR2^{KD}$ [a]	0.999 ± 0.014	2113	0.500 ± 0.030	51
HeLaP4 TRN-$SR2^{SCR}$ [a]	1.003 ± 0.014	2180	0.498 ± 0.023	76
HeLaP4 LEDGF/$p75^{KD}$ [a]	1.173± 0.017	1632	0.679 ± 0.029	63
HeLaP4 LEDGF/$p75^{Flox}$ [a]	1.228 ± 0.016	1968	0.623 ± 0.027	52

[a] VSV-G pseudotyped $HIV_{IN\text{-}eGFP}$ viral particles were used for infection of the different cell types.

[b] The different cell lines are explained in Fig. 2 and 3. (KD = knock down; SCR = scrambled)

[c] Each individual viral complex was localized and fitted with a 2D Gaussian curve, and the integrated intensity was calculated in each *z*-slice (See also Supplementary Fig. S2). Typically data were collected from ~30 different cells corresponding to at least 1000 detected complexes per experiment, except for $CD4^+$ T cells. Each experiment was at least performed twice and data from a representative experiment are shown. The geometric mean of the intensity data is shown, ± represents the back-transformed standard error of the mean (Supplementary Fig. S4).

[d] N is the number of single viral complexes used to calculate the geometric mean of the intensity data.

[e] For the imaging of $HIV^{Env}_{IN\text{-}eGFP}$, with a wild-type HIV envelope, in C8166 T cells and $CD4^+$ T cells a higher excitation power was used. The intensity distribution of complexes in C8166 T cells and $CD4^+$ T cells is not log-normal distributed. Therefore the median of the intensity data is shown, ± represents the standard error of the median (MAD/N^0.5).

Table S3. FRET ratios of $HIV_{IN\text{-}mTFP1+IN\text{-}mVenus}$ or $HIV_{IN\text{-}W131A\text{-}mTFP1+IN\text{-}W131A\text{-}mVenus}$ Viral Complexes. Related to Figure 2, 3 and 4.

$HIV_{IN\text{-}mTFP1+IN\text{-}mVenus}$ (a1)	Cytoplasm		Nucleus	
**Cell type(b)**	**FRET ratio(c)**	**N(d)**	**FRET ratio(c)**	**N(d)**
HeLaP4 (a1)	1.26 ± 0.01	1064	1.44 ± 0.07	122
C8166 T cell line (a2)	1.26 ± 0.02	439	1.39 ± 0.05	30
$CD4^+$ T cell (a2)	1.27 ± 0.04	56	1.42 ± 0.08	9
HeLaP4 TRN-SR2^{KD} (a1)	1.30 ± 0.01	1986	1.39 ± 0.03	111
HeLaP4 TRN-SR2^{SCR} (a1)	1.27± 0.01	1557	1.39 ± 0.04	152
HeLaP4 LEDGF/p75^{KD} (a1)	1.27± 0.01	1906	1.27 ± 0.02	283
HeLaP4 LEDGF/p75^{Flox} (a1)	1.26± 0.01	714	1.40 ± 0.06	62
HeLaP4 LEDGF/p75^{BC} (a1)	1.30± 0.01	1531	1.50 ± 0.05	134
HeLaP4 LEDGF/p75^{D366N} (a1)	1.25± 0.01	767	1.28 ± 0.03	89
HeLaP4 LEDGF/p75$^{93\text{-}530}$ (a1)	1.29± 0.01	1055	1.40 ± 0.02	115
HeLaP4 LEDGF/p75^{AT} (a1)	1.24± 0.01	2755	1.40 ± 0.02	204
HeLaP4 LEDGF/p75$^{325\text{-}530}$ (a1)	1.30± 0.02	443	1.41 ± 0.05	47
HeLaP4 LEDGF/p75$^{325\text{-}530;D366N}$ (a1)	1.31± 0.01	445	1.33 ± 0.03	71
**$HIV_{IN\text{-}W131A\text{-}mTFP1+IN\text{-}W131A\text{-}mVenus}$ (e)**	**Cytoplasm**		**Nucleus**	
**Cell type**	**FRET ratio(c)**	**N(d)**	**FRET ratio(c)**	**N(d)**
HeLaP4	1.29 ± 0.01	747	1.34 ± 0.04	69

(a) (1) VSV-G pseudotyped or (2) HIV enveloped $HIV_{IN\text{-}mTFP1+IN\text{-}mVenus}$ viral particles were used for infection of the different cell types.

(b) The different cell lines are explained in Fig. 2, 3 and 4 and Supplementary Fig. S8 (KD= knockdown; SCR= scrambled, BC= back-complemented).

(c) Each individual viral complex was localized and fitted with a 2D Gaussian curve, the FRET ratio was calculated using Eq. 1. Typically data were collected from ~50-100 different cells corresponding to an average of ~500-3000 viral complexes. Shown here is the mean FRET ratio of all localized viral complexes (N). ± represents the standard error of the mean (SEM).

(d) N is the number of single viral complexes used to calculate the average FRET ratio.

(e) Introduction of a W131A mutation interferes with the binding of IN to LEDGF/p75.

Table S4. Intensity of $HIV_{IN\text{-}eGFP,IN\text{-}HA}$ Viral Complexes. Related to Supplementary Figure S7.

$HIV_{IN\text{-}eGFP,IN\text{-}HA}$ [(a)]	Cytoplasm		Nucleus	
**HeLaP4**	**Intensity[(b)]**	**N[(c)]**	**Intensity[(b)]**	**N[(c)]**
IN-eGFP	0.940 ± 0.057	232	0.405 ± 0.035	20
IN-HA	0.982 ± 0.052	232	0.488 ± 0.065	20

[(a)] VSV-G pseudotyped $HIV_{IN\text{-}eGFP,IN\text{-}HA}$ viral particles were used for infection of HeLaP4 cells.

[(b)] Each individual viral complex was localized and fitted with a 2D Gaussian curve, and the integrated intensity was calculated in each *z*-slice (See also Supplementary Fig. S2). Typically data were collected from ~40 different cells corresponding to at least 200 detected complexes per experiment, containing both IN-eGFP and IN-HA. The experiment was performed twice and data from a representative experiment are shown. The geometric mean of the intensity data is shown, ± represents the back-transformed standard error of the mean (Supplementary Fig. S4).

[(c)] N is the number of single viral complexes used to calculate the geometric mean of the intensity data.

Table S5. Intensity of $HIV_{IN\text{-}eGFP}$ Viral Complexes after Addition of Raltegravir, Elvitegravir or LEDGINs during Infection. Related to Figure 5 and 6.

**$HIV_{IN\text{-}eGFP}$[(a)]**	**Cytoplasm**		**Nucleus**	
**Inhibitor**	**Intensity[(b)]**	**N[(c)]**	**Intensity[(b)]**	**N[(c)]**
DMSO	1.001 ± 0.017	1353	0.431 ± 0.040	39
RAL	0.944 ± 0.013	1978	0.433 ± 0.021	80
EVG	0.908 ± 0.015	1698	0.407 ± 0.019	98
LEDGINs	0.936 ± 0.014	1549	0.446 ± 0.031	57

[(a)] VSV-G pseudotyped $HIV_{IN\text{-}eGFP}$ viral particles were used for infection of HeLaP4 in the presence of DMSO, RAL (0.6 µM), EVG (0.2 µM) or LEDGINs (CX14442, 45 µM).

[(b)] Each individual viral complex was localized and fitted with a 2D Gaussian curve, and the integrated intensity was calculated in each *z*-slice (See also Supplementary Fig. S2). Typically data were collected from ~30 different cells corresponding to at least 1000 detected complexes per experiment. Each experiment was at least performed twice and data from a representative experiment are shown. The geometric mean of the intensity data is shown, ± represents the back-transformed standard error of the mean (Supplementary Fig. S4).

[(c)] N is the number of single viral complexes used to calculate the geometric mean of the intensity data.

Table S6. Intensity of $HIV_{IN\text{-}eGFP}$ Viral Complexes at Different Time Points post Infection. Related to Figure 5.

$HIV_{IN\text{-}eGFP}$ [a]	Cytoplasm		Nucleus	
**Time (h)**	**Intensity[b]**	**N[c]**	**Intensity[b]**	**N[c]**
0	0.891 ± 0.705	353	/	0
2	1.013 ± 0.735	1345	0.451 ± 0.115	8
4	1.006 ± 0.691	792	0.535 ± 0.208	19
6	0.897 ± 0.728	604	0.509 ± 0.279	45
8	1.168 ± 1.205	427	0.566 ± 0.251	41
12	1.376 ± 1.625	300	0.705 ± 0.534	34
24	1.071 ± 1.162	207	0.582 ± 0.351	38

[a] VSV-G pseudotyped $HIV_{IN\text{-}eGFP}$ viral particles were used for synchronized infection. The zero time point (0 h) represents 2 h of infection at 16°C, which was followed by a temperature shift to 37°C. After 2 h of incubation at 37°C (2 h time point), cells were washed and further incubated to reach a total infection time of 24h.

[b] Each individual viral complex was localized and fitted with a 2D Gaussian curve, and the integrated intensity was calculated in each *z*-slice (See also Supplementary Fig. S2). Typically data were collected from ~30 different cells. Each experiment was at least performed twice and data from a representative experiment are shown. The geometric mean of the intensity data is shown; ± represents the back-transformed standard deviation.

[c] N is the number of single viral complexes used to calculate the geometric mean of the intensity data.

Table S7. Intensity of $HIV_{IN\text{-}eGFP}$ Viral Particles or Viral Complexes and FRET ratios of $HIV_{IN\text{-}mTFP1+IN\text{-}mVenus}$ Viral Complexes after Addition of LEDGINs during Production. Related to Figure 5.

$HIV_{IN\text{-}eGFP}$ [(a1)]	Particles		Cytoplasm		Nucleus	
**Inhibitor**	**Intensity[(b)]**	**N[(d)]**	**Intensity[(b)]**	**N[(d)]**	**Intensity[(b)]**	**N[(d)]**
DMSO	1.844 ± 0.044	428	1.068 ± 0.027	862	0.500 ± 0.026	55
LEDGINs	1.790 ± 0.035	778	1.487 ± 0.032	1061	0.634 ± 0.160	8

$HIV_{IN\text{-}mTFP1+IN\text{-}mVenus}$ [(a2)]	Cytoplasm		Nucleus	
**Inhibitor**	**FRET ratio[(c)]**	**N[(d)]**	**FRET ratio[(c)]**	**N[(d)]**
DMSO	1.26 ± 0.01	512	1.44 ± 0.07	57
LEDGINs	1.39 ± 0.03	929	1.44 ± 0.09	64

[(a)] (1) VSV-G pseudotyped $HIV_{IN\text{-}eGFP}$ (2) and VSV-G pseudotyped $HIV_{IN\text{-}mTFP1+IN\text{-}mVenus}$ viral particles produced in the presence of DMSO or LEDGINs (CX14442, 1.2 µM) were used for infection of HeLaP4 cells.

[(b)] Each individual viral particle or complex was localized and fitted with a 2D Gaussian curve, and the integrated intensity was calculated in each *z*-slice (Supplementary Fig. S2). Typically data were collected from ~30 different cells corresponding to at least 1000 detected complexes per experiment. Each experiment was at least performed twice and data from a representative experiment are shown. The geometric mean of the intensity data is shown, ± represents the back-transformed standard error of the mean (Supplementary Fig. S4).

[(c)] Each individual viral complex was localized and fitted with a 2D Gaussian curve, the FRET ratio was calculated using Eq. 1. Typically data were collected from ~50-100 different cells corresponding to an average of ~500-3000 viral complexes. Shown here is the mean FRET ratio of all localized viral complexes (N). ± represents the standard error of the mean (SEM).

[(c)] N is the number of single viral complexes used to calculate the geometric mean of the intensity data or the FRET ratio.

Table S8. FRET ratios of $HIV_{IN\text{-}mTFP1+IN\text{-}mVenus}$ Viral Complexes after Addition of LEDGINs during Infection of LEDGF/p75^{KD} cells. Related to Figure 6.

$HIV_{IN\text{-}mTFP1+IN\text{-}mVenus}$ [(a)]	Cytoplasm		Nucleus	
**Inhibitor**	**FRET ratio[(b)]**	**N[(c)]**	**FRET ratio[(b)]**	**N[(c)]**
DMSO	1.27 ± 0.01	425	1.24 ± 0.04	40
LEDGINs	1.28 ± 0.01	373	1.41 ± 0.03	49

[(a)] VSV-G pseudotyped $HIV_{IN\text{-}mTFP1+IN\text{-}mVenus}$ viral particles were used for infection of HeLaP4 LEDGF/p75^{KD} cells in the presence of DMSO or LEDGINs (CX14442, 45 µM).

[(b)] Each individual viral complex was localized and fitted with a 2D Gaussian curve, the FRET ratio was calculated using Eq. 1. Typically data were collected from ~20-50 different cells corresponding to an average of ~500 viral complexes. Shown here is the mean FRET ratio of all localized viral complexes (N). ± represents the standard error of the mean (SEM).

[(c)] N is the number of single viral complexes used to calculate the average FRET ratio.

SUPPLEMENTARY FIGURES

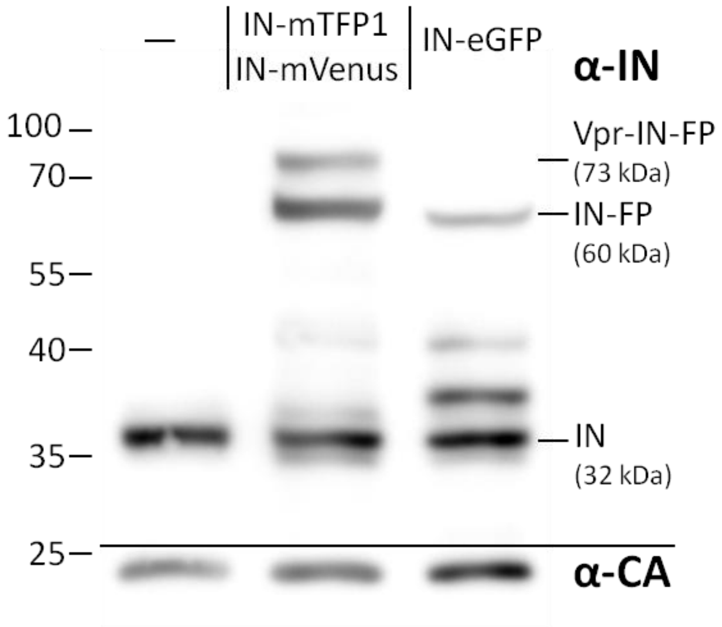

Figure S1. Western blot showing Incorporation of (Vpr)-IN-FP into HIV-1 Viral Particles. Related to Figure 1.

Lysed viral particles were analyzed using Western blot with antibodies against IN (top) and CA (bottom). Viruses are produced as in Figure 1A, the Vpr-transincorporated IN-FP is shown and '-' represents the absence of transincorporation.

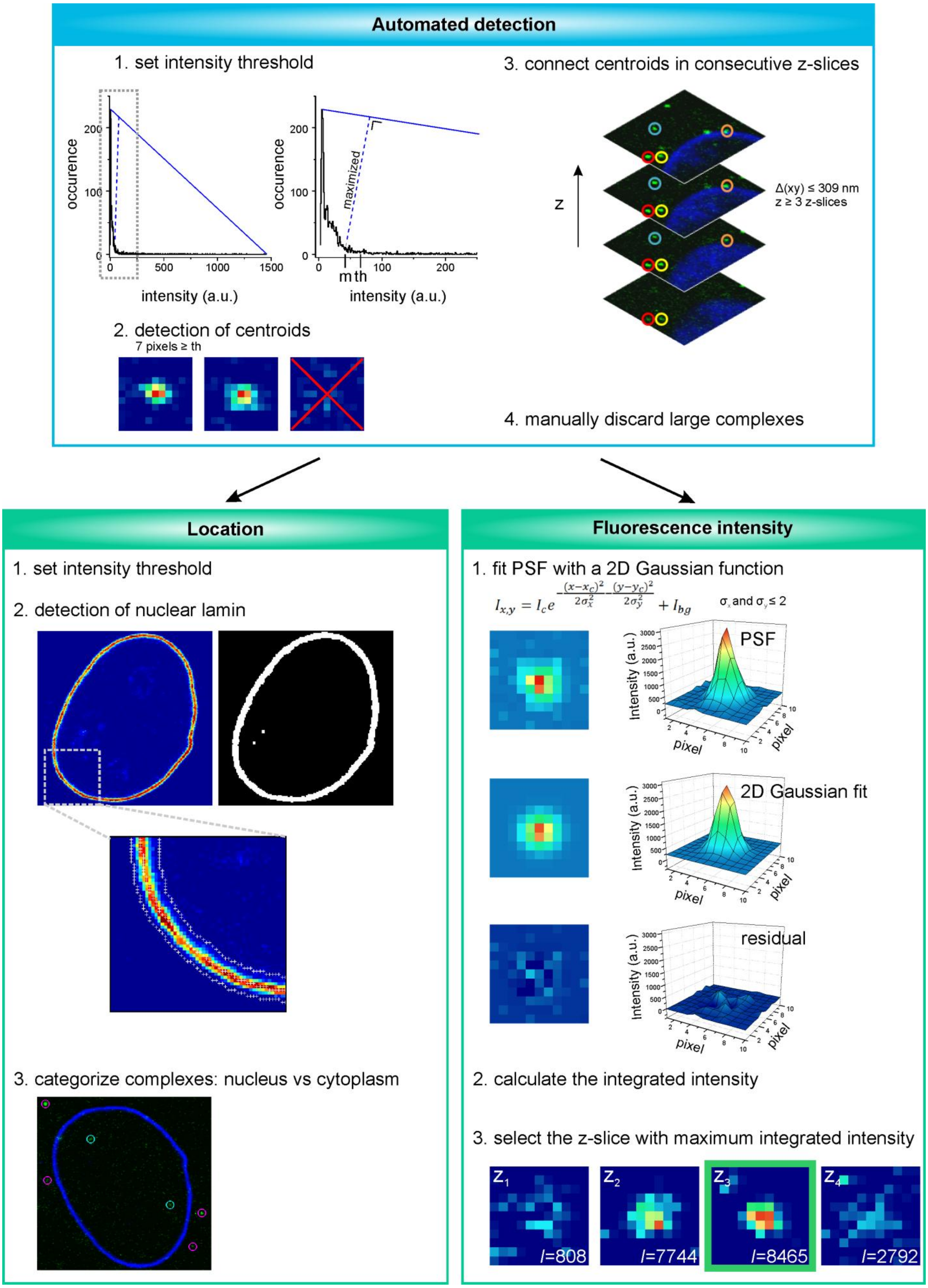

Automated detection
1. set intensity threshold
occurence
intensity (a.u.)
maximized
m th
2. detection of centroids
7 pixels ≥ th
3. connect centroids in consecutive z-slices
z
Δ(xy) ≤ 309 nm
z ≥ 3 z-slices
4. manually discard large complexes
Location
1. set intensity threshold
2. detection of nuclear lamin
3. categorize complexes: nucleus vs cytoplasm
Fluorescence intensity
1. fit PSF with a 2D Gaussian function
$I_{x,y} = I_c e^{-\frac{(x-x_c)^2}{2\sigma_x^2}-\frac{(y-y_c)^2}{2\sigma_y^2}} + I_{bg}$
σ_x and $\sigma_y \leq 2$
PSF
2D Gaussian fit
residual
Intensity (a.u.)
pixel
2. calculate the integrated intensity
3. select the z-slice with maximum integrated intensity
Z_1 I=808
Z_2 I=7744
Z_3 I=8465
Z_4 I=2792

Figure S2. Workflow of the Automated Image Analysis. Related to Figure 1.

The automatic detection, localization and intensity calculation of each IN-eGFP complex was performed using a homemade MatLab routine (The MathWorks, Inc.). Top: (1) The threshold (th) for the detection of centroids of each PIC is calculated using the triangle algorithm[1], which is based on the pixel intensities histogram. A triangle is defined by the height, the dynamic range and the point in the histogram by which the altitude of the triangle is maximized. To the corresponding intensity (m) the double of the mean image intensity of each *z*-slice was added, which results in the threshold (th). (2) Only centroids with more than 7 pixels above background are detected. (3) The centroids are assigned to be part of a single IN-eGFP complex (indicated with the same coloured circle) if they are observed in at least 3 consecutive *z*-slices at a maximum distance of 3 pixels. (4) Large aggregates are manually removed. Left: (1) The threshold (th) for the detection of the nuclear lamin is calculated using the triangle algorithm. (2) The middle, the inner and outer of the nuclear lamin (indicated by +++) is automatically detected based on intensity thresholding. The black and white image shows the assignment of the nuclear lamin. (3) Based on the nuclear lamin (blue), complexes are assigned to the cytoplasm (magenta) or nucleus (cyan). Right: (1) The point spread function of each spot is fitted with a 2D Gaussian function using the least-mean-square method, the residual after fitting is shown as well. Complexes with width (σ_x and σ_y) larger than 2 pixels are excluded since these are assumed to be aggregates. (2) The integrated intensity of this function is calculated in each *z*-slice. (3) The *z*-slice and corresponding highest intensity is selected for further usage, since in this *z*-slice the complex is in focus.

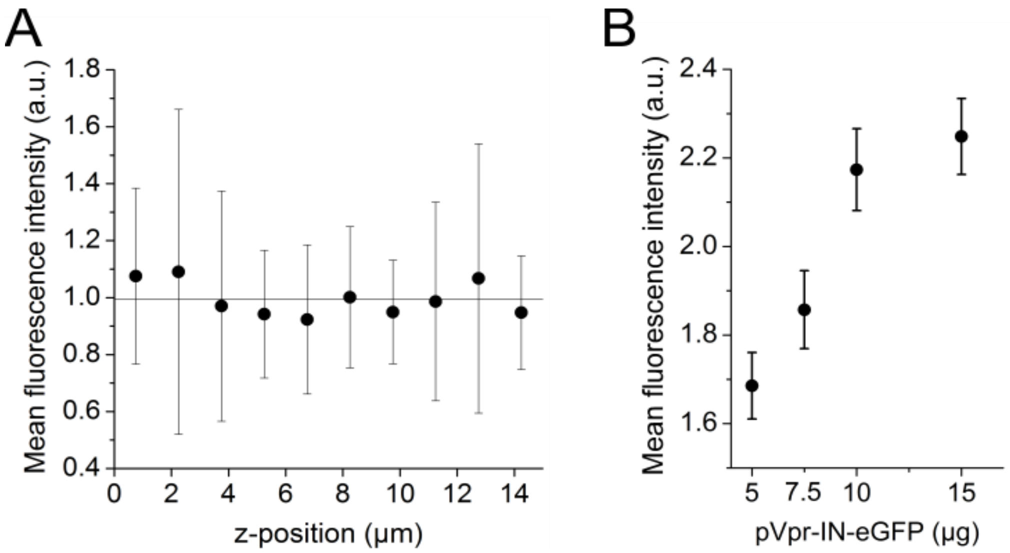

Figure S3. Validation of Image Acquisition and Intensity Calculations. Related to Figure 1 and Supplementary Table S1.

(A) The invariable mean fluorescence intensity of 100 nm TetraSpeckTM Microspheres embedded in agarose at different *z*-positions excludes any effect of *z*-position-dependent spherical aberrations on the intensity calculation. The horizontal line represents mean intensity of all microspheres and error bars represent the standard deviation within each bin (1.5 µm). (B) Mean fluorescence intensity of $HIV_{IN\text{-}eGFP}$ particles produced with different amounts of pVpr-IN-eGFP plasmid. Single particle intensity analyses shows that an increased amount of IN-eGFP corresponds to an increased fluorescence intensity of the $HIV_{IN\text{-}eGFP}$ particles, proving that the intensity calculation can discriminate different amounts of labeled IN. Error bars represent the standard error of the mean (SEM).

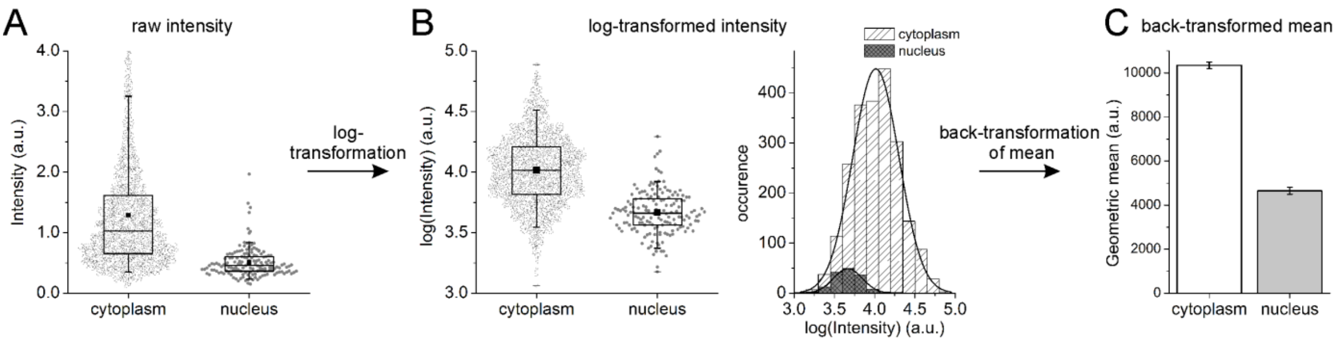

Figure S4. Log-normal Distribution of Fluorescence Intensity of $HIV_{IN\text{-}eGFP}$. Related to Figure 2.

(A) Raw fluorescence intensity of $HIV_{IN\text{-}eGFP}$ complexes in infected HeLaP4 cells in the cytoplasm (black circle) and in the nucleus (grey circle), obtained by our automated image analysis tool. Box-plot whiskers represent 5th and 95th percentile, the median value ($\tilde{x}_{cyt}$=10,321; $\tilde{x}_{nuc}$=4,586) is represented by the line within the box, and the square depicts the mean ($\bar{x}_{cyt}$=12,901; $\bar{x}_{nuc}$=5,089). (B) By log-transforming the raw intensity values, the box plot (left) and histogram (right) show that the common logarithm of fluorescence intensity of $HIV_{IN\text{-}eGFP}$ is normally distributed (Kolmogorov-Smirnov test: p-value < 0.05), hence the fluorescence intensity is log-normal distributed ($\tilde{x}_{log(cyt)}$=4.01; $\tilde{x}_{log(nuc)}$=3.66; $\bar{x}_{log(cyt)}$=4.01; $\bar{x}_{log(nuc)}$=3.67). (C) The mean of the log-transformed intensity is back-transformed to obtain the geometric mean intensity ($\bar{x}^*_{cyt}$=10,348; $\bar{x}^*_{nuc}$=4,649). The error bars were calculated as follows[2]: the standard error of the mean (SEM) of the log-transformed intensity (B) was back-transformed into SEM*. Since SEM* represents the multiplicative SEM, the range of the positive error bar corresponds to $\bar{x}^*$ x SEM*, while the negative error bar corresponds to $\bar{x}^*$/ SEM*.

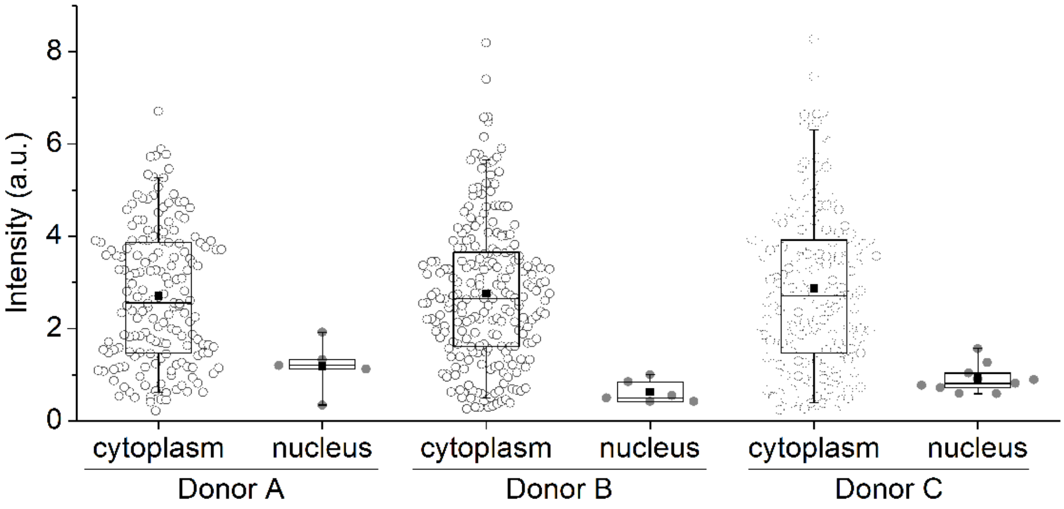

Figure S5. Decrease in fluorescence intensity in the nucleus of primary CD4$^+$ T cells of different donors*.* Related to Figure 2.

Fluorescence intensity of HIV complexes (HIV$^{Env}_{IN\text{-}eGFP}$) in infected primary CD4$^+$ T cells of different donors (A, B, C) in the cytoplasm and in the nucleus. Box-plot whiskers represent 5th and 95th percentile, the median value is represented by the line within the box, and the square depicts the mean.

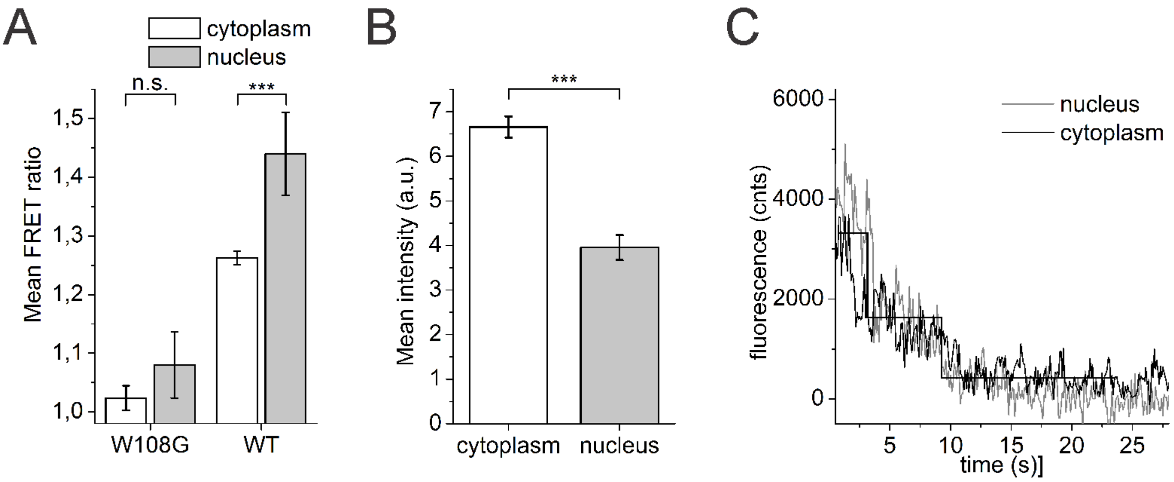

Figure S6. Altered Nuclear FRET and Fluorescence Intensity of HIV IN Oligomers are not due to the Nuclear Environment. Related to Figure 2.

(A) Macromolecular crowding, a term used to describe the effect of high total volume occupancy by a macromolecular complex upon the behavior of each macromolecular species[3] is a feature that often takes place inside the nucleus. Crowding forces, which are higher in the nucleus because of the high macromolecular content, vastly increase the association constants of intermolecular interactions[4]. Macromolecular crowding in the nucleus could stimulate intermolecular interactions between IN subunits, resulting in an increased FRET signal. However, the constant FRET-ratio (around unity) of $HIV_{IN\text{-}W108G\text{-}mTFP1+IN\text{-}W108G\text{-}mVenus}$ complexes, carrying an IN mutant known to reduce IN dimerization and higher order oligomerization[5], in the cytoplasm (white) and nucleus (grey) in HeLaP4 cells in contrast to the increase in FRET ratio with WT IN, shows that macromolecular crowding does not explain the increased FRET ratio in the nucleus. (B) Geometric mean of fluorescence intensity in the cytoplasm (white) or nucleus (grey) in HeLaP4 cells infected with VSV-G pseudotyped $HIV_{IN\text{-}mTFP1}$. Error bars represent standard error of the mean (SEM). $HIV_{IN\text{-}mTFP1}$ and $HIV_{IN\text{-}eGFP}$ (Fig. 2B) complexes show a similar decrease in fluorescence intensity upon nuclear entry, indicating that the intensity decrease is independent of fluorescent protein variants. Since $HIV_{IN\text{-}mTFP1}$ was produced with only 5 µg of Vpr-IN-mTFP1 plasmid, it furthermore shows that the intensity decrease is not related to the amount of IN-FP initially present in the particle. (C) Stepwise single-molecule photobleaching of $HIV_{IN\text{-}mVenus}$ exhibits two-step photobleaching in the cytoplasm (gray) and in the nucleus (black). Since the drop in intensity upon stepwise photobleaching is identical for IN-mVenus complexes in the nucleus and the cytoplasm, we argue that different environments (nucleus or cytoplasm) have no influence on the photophysical behavior of the fluorescent proteins and accordingly do not cause the reduced nuclear fluorescence intensity.

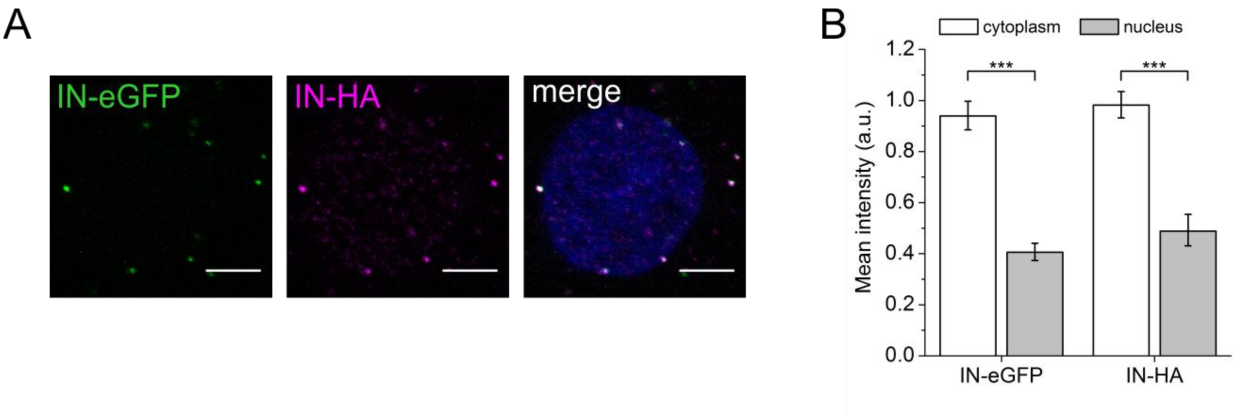

Figure S7. Decrease in Fluorescence Intensity in the Nucleus of HeLaP4 cells Infected with $HIV_{IN\text{-}eGFP,IN\text{-}HA}$. Related to Figure 2 and Supplementary Table S4.

(A) In VSV-G pseudotyped $HIV_{IN\text{-}eGFP,IN\text{-}HA}$, IN-eGFP is provided using the Vpr-transincorporation technique, while IN-HA is encoded by the molecular clone pNL4-3.Luc.R^-.E^-.IN-HA. Only complexes containing IN-eGFP (green) and IN-HA, labeled with an Alexa 647 conjugated primary HA antibody (magenta), were analyzed. Scale bars represent 5 µm. (B) The fluorescence intensity of eGFP or Alexa 647 in $HIV_{IN\text{-}eGFP,IN\text{-}HA}$ complexes in the cytoplasm (white) and in the nucleus (grey) of HeLaP4 cells. (****p*-value < 0.001)

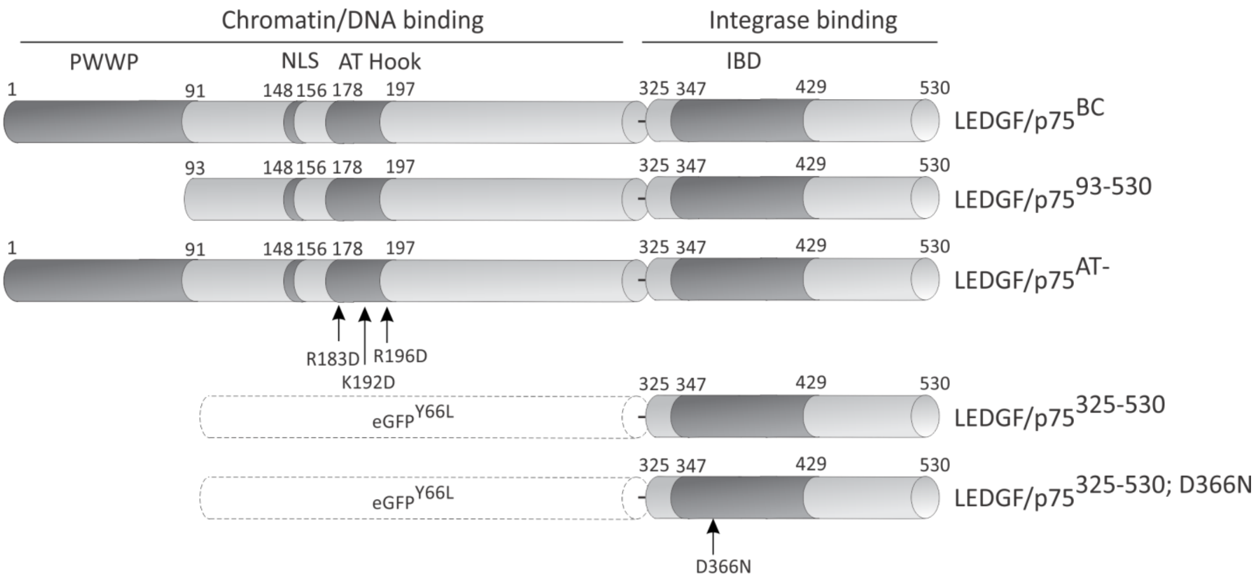

Figure S8. Schematic Representation of the LEDGF/p75 Domain Structure and the DNA-binding Mutants. Related to Figure 4.

To interfere with the chromatin binding, several truncations of LEDGF/p75 were stably expressed in LEDGF/p75 KD cells. These include a PWWP deletion mutant (LEDGF/p75$^{93\text{-}530}$), a triple mutation (R183D, K192D and R196D) of the AT-hook like motifs (LEDGF/p75^{AT-}), a deletion of the complete N-terminal domain (LEDGF/p75$^{325\text{-}530}$) and its IN interaction mutant D366N (LEDGF/p75$^{325\text{-}530;\ D366N}$). The two latter LEDGF/p75 truncations are fused to the C-terminus of a non-fluorescent eGFP^{Y66L} to increase protein stability. To control for overexpression we also expressed wild-type LEDGF/p75 in LEDGF/p75 KD cells to generate LEDGF/p75^{BC}. The N-terminal domain of LEDGF/p75 is involved in chromatin/DNA binding and the C-terminal domain is involved in HIV IN binding through the integrase binding domain (IBD). BC = back-complemented, PWWP = Pro-Trp-Trp-Pro domain.

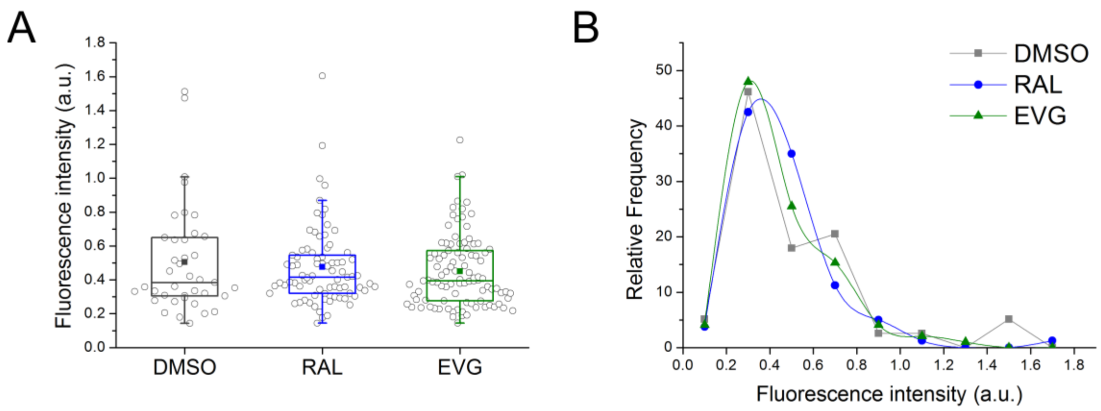

Figure S9. Fluorescence Intensity Distribution of Nuclear HIV$_{\text{IN-eGFP}}$ Complexes with and without Treatment with INSTIs Raltegravir and Elvitegravir. Related to Figure 5 and Supplementary Table S5. (A) Fluorescence intensity of HIV$_{\text{IN-eGFP}}$ complexes in the nucleus of HeLaP4 cells treated with DMSO, RAL (0.6 µM) or EVG (0.2 µM) during infection. (B) The nuclear HIV$_{\text{IN-eGFP}}$ complexes were binned according to fluorescence intensity (0.2 a.u.), the relative frequency per bin is shown. Since the intensity distribution does not change upon treatment with INSTIs, we can conclude that the labeling density does not determine the functionality of the nuclear complexes.

SUPPLEMENTARY METHODS

Plasmids and siRNA transfections

The following reagents were obtained through the NIH AIDS Reagent Program, Division of AIDS, NIH: pNL4-3 (Cat #114) from Dr. Malcolm Martin [6], pNL4-3.Luc.R^{-}.E^{-} (Cat #3418) from Dr. Nathaniel Landau [7]. The Vpr-IN-eGFP construct is described in Albanese *et al.* [9] and both Vpr-IN-mTFP1 and Vpr-IN-mVenus plasmids are described in Borrenberghs *et al.* [10]. The W131A mutation was introduced in the pVpr-IN-FP constructs *via* site directed ligase-independent mutagenesis (SLIM) with the following primer 5'-GAAGGCCGCCTGCGCGTGGCCGGCATC. The original template was digested with DpnI. The HA-tag was introduced in pNL4-3.Luc.R^{-}E^{-} *via* SLIM [11] with the following primers: F_T 5'-TACCCATACGATGTTCCAGATTACGCTTAACACATGGAAAAGATTAGTAAAACACC, F_S 5'-TAAC ACATGGAAAAGATTAGTAAAACACC,
R_T 5'-AGCGTAATCTGGAACATCGTATGGGTAATCCTCATCCTGTCTACTTGCCAC,
R_S 5'-ATCCTCATCCTGTCTACTTGCCAC, the original template was digested with DpnI. $HIV_{IN\text{-}eGFP,IN\text{-}HA}$ was produced in the same way as $HIV_{IN\text{-}eGFP}$, but now using pNL4-3.Luc.R^{-}E^{-}.IN-HA. SIV-based vector plasmids (pGAE_CAG-eGFP-WPRE) were a kind gift of D. Nègre (Laboratoire de Vectorologie Rétrovirale et Thérapie Génique, INSERM U412, IFR 74, Ecole Normale Supérieure de Lyon, Lyon, France). A lentiviral vector carrying the i.e. hCMV promoter driving a Zeocin resistance gene and a LEDGF/p75 specific miRNA-based shRNA was described earlier [12]. In this transfer plasmid we introduced two loxP-sites (ATAACTTCGTATA ATGTATGC TATACGAAGTTAT) [13] to allow Cre-specific recombination, one before and one after the miRNA-based hairpin. Cre-recombinase was provided transiently as virus-like particles (VLP) (ASLV/VLP_Cre) as decribed earlier [14] resulting in LEDGF/p75^{Flox} cells. For the following pGAE-SFFV_LEDGF BC_Ires_BsdR_WPRE, pGAE-SFFV_LEDGF D366N BC_Ires_BsdR_WPRE and pGAE-SFFV_dPWWP_Ires_BsdR_WPRE constructs, we used pGAE-SFFV_CBX Δ325 BC_Ires_BsdR_WPRE [15] as backbone and replaced the CBX Δ325 by the corresponding LEDGF/p75 variant. The LEDGF/p75 variants were digested from the corresponding pLNC_LEDGF BC-Ires-Bsd, pLNC_LEDGF BC D366N-Ires-Bsd or pLNC_ΔN_{93} LEDGF BC-Ires-Bsd plasmid. AT-hook and NLS LEDGF/p75 variants were constructed by amplification of LEDGF/p75 with 5'-AAAAAAAGATCTGTATGACTCGCGATTTCAAACCTG and 5'-AACTCACAACGTGGCACTGG as sense and antisense primers, respectively. The amplicon was digested by BglII and ApaI and cloned in pGAE-SFFV_CBX Δ325 BC_Ires_BsdR_WPRE [15] resulting in pGAE-SFFV_LEDGF NLS BC_Ires_BsdR_WPRE and pGAE-SFFV_LEDGF AT1+2 BC_Ires_BsdR_WPRE. To create pGAE-SFFV eGFP^{Y66L} Δ325 BC_Ires_BsdR_WPRE and pGAE-SFFV eGFP^{Y66L} Δ325 D366N BC_Ires_BsdR_WPRE, the CBX gene in pGAE-SFFV_CBX Δ325 BC_Ires_BsdR_WPRE was replaced by a gene block fragment (Integrated DNA technology, Belgium) encoding the non-fluorescent eGFP (Y66L) flanked by a BglII and XhoI restriction site. Both the gene fragment and the pGAE-SFFV_CBX Δ325 BC_Ires_BsdR_WPRE are digested with BglII and XhoI. The SIV-based lentiviral vectors were produced as previously described [16]. Briefly, VSV-G pseudotyped lentiviral vectors (LV) were produced by triple polyethylenimine (PEI)-mediated transfection of 293T cells using per 10 cm dish 5 µg of the VSV-G plasmid, 15 µg of a packaging plasmid and 15 µg of the transfer plasmid carrying the reporter gene flanked by two long terminal repeats. Two and three days after transfection, the supernatant was harvested, filtered with 0.45 µm pore-size syringe filters (Sartorius) and concentrated by ultrafiltration (Vivaspin, MWCO 50K, Merck,

Overijse, Belgium), aliquoted and stored at -80°C. HeLaP4 cells were next transduced with LV with a multiplicity of infection (MOI) < 1. After 72 h, the medium was replaced by medium with blasticidin (10 µg/ml, Invivogen) and Zeocin (200 µg/ml, Invivogen) to select transduced cells. Following selection, cells were cultured at 37°C in a humidified atmosphere containing 5% CO_2. TRN-SR2^{KD} cells were generated by transduction with a LV expressing a shRNA targeting TRN-SR2 mRNA (shTR3, Sigma, clone-id: NM_12470.2-867s21c1). A control cell line was established using a control vector expressing a scrambled shRNA referred to as shSCR (Sigma, product number SHC002). These lentiviral transfer plasmids were a kind gift from Dr. R. Hoeben (Leiden University Medical Center, The Netherlands). The shRNA expressing transfer plasmids are based on the pLKO.1 plasmid (Sigma) containing a puromycin resistance cassette.

Eukaryotic cell culture

HEK293T cells (ATCC CRL-11268) were cultured at 37°C in a 5% CO_2 humidified atmosphere in Dulbecco's modified Eagle medium with GlutaMAX (DMEM, Life Technologies Europe, Merelbeke, Belgium) supplemented with 5% (v/v) fetal bovine serum (FBS, Life Technologies) and 50 µg/ml Gentamicin (Life Technologies). For HeLaP4 (a kind gift from Pierre Charneau, Institut Pasteur, Paris, France) and HeLaP4 LEDGF/p75^{Flox} cells the previous medium was additionally supplemented with 500 µg/mL geneticin (Gibco BRL). The following selective antibiotics were added to the growth medium of HeLaP4-derived cells: 100 µg/ml zeocin (Invivogen) for LEDGF/p75^{KD} cells , 100 µg/ml zeocin and 10 µg/ml blasticidin (Invivogen) for LEDGF/p75 transduced cells (LEDGF/p75^{BC}, LEDGF/p75^{D366N}, LEDGF/p75$^{93\text{-}530}$, LEDGF/p75^{AT-}, LEDGF/p75$^{325\text{-}530}$ and LEDGF/p75$^{325\text{-}530,\ D366N}$) and 1 µg/ml puromycin (Invivogen) for TRN-SR2^{KD} and TRN-SR2^{SCR} cells.

Immunocytochemistry

For confocal microscopy, infected cells were permeabilized with 0.1% (v/v) Triton X-100 (Sigma-Aldrich) in PBS and immunostained for the nuclear lamina with lamin AC antibody (1/500 dilution, sc-7292, Santa Cruz Biotechnology) and secondary goat anti-mouse IgG Alexa-Fluor 633 conjugate (1/500 dilution, Life technologies) diluted in blocking buffer (1% BSA (Sigma-Aldrich) and 0.1% Tween-20 (v/v) (A4974, Applichem) in PBS). For wide-field imaging, cells were immunostained for the epidermal growth factor receptor (EGFR) in the plasma membrane (1/250 dilution, AB-5, Calbiochem, Merck, Overijse, Belgium), followed by permeabilization with 0.1% (v/v) Triton X-100 in PBS and immunostained for the nuclear lamina with lamin AC antibody (1/1000 dilution, sc7293, Santa Cruz biotechnology, Heidelberg, Germany). After staining with a secondary goat anti-mouse Atto647N antibody (1/500 dilution, Molecular Probes, Invitrogen), cells were kept in PBS before imaging. CD4$^+$ T cells are additionally stained with DAPI (1/1000 dilution, Invitrogen). For experiments with HIV$_{IN\text{-}eGFP,IN\text{-}HA}$, the HA-tag was immunostained with HA-Tag (6E2) Mouse mAb Alexa Fluor 647 conjugate (1/500 dilution, #3444, Cell Signaling Technology) and nuclear DNA was stained with DAPI (1/1000 dilution).

To verify expression and knock-down of LEDGF/p75 in their respective HeLaP4-derived cell lines, 3×10^4 cells were seeded per well in poly-D-lysine (0.1 mg/ml) coated 8-well chambered cover glass and incubated overnight. Next, cells were fixed with 4% (v/v) paraformaldehyde, permeabilized with 0.1% (V/V) Triton X-100 in PBS and immunostained for LEDGF/p75 using a polyclonal anti-LEDGF antibody

(1/200 dilution, A300-848a, Bethyl Laboratories, Montgomery, TX) and goat anti-rabbit ATTO647N secondary antibody (1/500 dilution, Molecular Probes, Invitrogen). DNA was stained using DAPI (1/1000 dilution).

C8166 T cell culturing and infection

C8166 T cells (NIH AIDS Reagent Program, Division of AIDS, NIH (Cat #404) from Dr. Robert Gallo [17]) were cultured at 37 °C in a 5% CO_2 humidified atmosphere in Roswell Park Memorial Institute (RPMI) 1640 medium with GlutaMAX and HEPES (72400, Life Technologies) supplemented with 15% (v/v) FBS. 2.5×10^5 C8166 cells were infected with $HIV^{Env}_{IN\text{-}eGFP}$ or $HIV^{Env}_{IN\text{-}mTFP1+IN\text{-}mvenus}$ (8 µg of p24 antigen). At 21 h post infection cells were washed with PBS, briefly incubated with trypsin (0.25% w/v), washed with growth medium and then plated in poly-D-lysine (0.1 mg/ml) coated 8-well chambers. The cells were allowed to adhere for 3 h at 37°C, to reach a total infection of 24 h. Next, cells were fixed with 4% (v/v) paraformaldehyde and immunostained.

PBMC isolation, $CD4^+$ T cell expansion and infection

Human peripheral blood mononuclear cells (PBMC) were purified from fresh buffy coats of anonymous voluntary donors from the University hospitals Gasthuisberg Leuven Blood Bank according to approved bioethical guidelines of our institute (S57175-IRB00002047) using Lymphoprep following the manufacturer's protocol (Axis-Shield PoC AS, Oslo, Norway) and frozen until further use. Thawed PBMC's were enriched for $CD4^+$ T cells using an anti-human CD3:8 bispecific monoclonal antibody (0.5 µg/ml, NIH AIDS Reagent Program, Division of AIDS, NIH (Cat #12277) from Drs. Johnson Wong and Galit Alter) during 5 days in RPMI supplemented with human IL2 (100 U/ml, PeproTech), MEM Non-Essential Amino Acids (1x) (Life Technologies) and 15% FBS (Life Technologies). Subsequently, 6×10^5 $CD4^+$ T cells were infected with $HIV^{Env}_{IN\text{-}eGFP}$ or $HIV^{Env}_{IN\text{-}mTFP1+IN\text{-}mVenus}$ (2.4 µg of p24-antigen). At 2 h post infection the cells were washed three times with PBS and further incubated for 19 hours. Next, the cells were plated in poly-D-lysine (0.4 mg/ml) coated 8-well chambers and allowed to adhere for 3 h at 37°C, to reach a total infection of 24 h. Next, cells were fixed with 4% (v/v) paraformaldehyde and immunostained.

Western blotting

Protein concentrations of viral particle lysates (10 µg/ml p24-antigen in 1%SDS (w/v) (Sigma-Aldrich)) or whole cell lysates (1×10^6 cells in 1% SDS (w/v)) were determined using a bicinchoninic acid (BCA) protein assay (BCA Protein Assay Kit, Thermo Scientific, Geel, Belgium). For Western blotting, 10 µg of the viral particle lysate or 30 µg of the cell lysate was directly loaded onto a 12.5% (w/v) sodiumdodecyl sulfate polyacrylamide gelelectrophoresis (SDS-PAGE) gel and electroblotted onto polyvinylidene difluoride membranes (PVDF, Bio-Rad laboratories, Nazareth Eke, Belgium). HIV IN was dectected with anti-HIV-1 IN (mouse monoclonal, 1/10000 dilution, IN-2 ab66645, Abcam, Cambridge, U.K.), HIV CA was detected with anti-HIV-1 CA (p24) (mouse monoclonal, 1/5000 dilution, AG3.0, NIH AIDS Reagent Program, Division of AIDS, NIH (Cat #4121) from Dr. Jonathan Allan). LEDGF/p75 was detected with rabbit anti-LEDGF/p75 (1/500 dilution, A300-848a, Bethyl Laboratories, Montgomery, TX). TRN-SR2 detection was done using monoclonal antibodies against TNR-SR2 (1/100 dilution, ab54353, Abcam). Equal loading of cell lysates was confirmed with β-tubulin antibody (1/1000 dilution, T-4026, Sigma) or GAPDH antibody (1/10000 dilution, ab9485, Abcam). Blots were subsequently stained with a horseradish

peroxidase-conjugated secondary antibody (Dako, Heverlee, Belgium) and detected by chemiluminescence (ECL$^+$, Amersham Bioscience, GE Healthcare Europe GmbH, Diegem, Belgium).

Single-cycle viral infectivity assay

HIV was produced as described earlier, but without trans-incorporation of Vpr-IN-FP. IN^{D64E} mutants were produced using pD64E. To determine viral infectivity, 1.5 x 10^4 HeLaP4 cells were seeded per well in a 96-well plate and infected the next day with a volume (equivalent to 300 ng of p24 antigen) of virus in triplicate in three dilutions. 24 h after infection the virus was removed and replaced by DMEM containing 5% FBS. At 72 h post-infection, cells were lysed in buffer (50 mM Tris, 200 mM NaCl, 0.2 % NP40 and 5 % glycerol) and luciferase activity was measured using fLuc assay reagent (ONE-Glo^{TM} Promega GMBH, Mannheim, Germany). Readouts were normalized for protein content as determined by the BCA-assay (BCA Protein Assay Kit, Thermo Scientific). Data are presented as relative infectivity compared to HIV; the standard deviation represents deviation of triplicates.

Quantitative PCR analysis of 2-LTR circles

Unlabeled VSV-G pseudotyped HIV was produced by seeding 6.5 × 10^6 HEK293T producer cells per 10 cm petri-dish in DMEM supplemented with 2% FBS. At a cell density of 90%, cells were transfected using branched PEI (70 µl, 10 µM, Sigma-Aldrich) [18] with 5 µg VSV-G and 20 µg pNL4-3.Luc.R^-.E^-. Six hours post transfection, the medium was replaced with pre-warmed DMEM (Life Technologies) supplemented with 2% FBS and 50 µg/ml gentamicin. Supernatant was collected 48 and 72 h post transfection, filtered through a 0.45-µm filter (Sartorius), and concentrated by ultrafiltration (Vivaspin, MWCO 50K). For the quantitative PCR analysis HeLaP4 cells were seeded (1 x 10^6 cells per well in 6-well plate) and infected the next day with this VSV-G pseudotyped HIV (5 µg p24 per well) in the presence of PF-03450074 (PF74, 10 µM), RAL (0.6 µM; 100 x IC_{50}), EVG (0.2 µM; 100 x IC_{50}) or DMSO. 3.5 h post infection, the cells were washed three times with PBS and incubated in fresh medium supplemented with the respective inhibitors. At 8, 24 and 48 h post infection cells were harvested and washed with PBS. Genomic DNA was extracted using the Sigma Mammalian genomic DNA Miniprep kit (Sigma-Aldrich, Diegem, Belgium). qPCR reactions contained 1x iQ Supermix (Bio-Rad Laboratories, Temse, Belgium), 300 nM forward primer (5'-GTGCCCGTCTGTTGTGTGACT-3'), 300 nM reverse primer (5'-CTTGTCTTCTTTGGGAGTGAATTAGC-3'),
200 nM 5'-FAM-TCCACACTGACTAAAAGGGTCTGAGGGATCTCT-Tamra-3' probe and 170 ng DNA, all samples were run in triplicate. A standard curve was generated and no-template controls were included. The qPCR was run on a LightCycler® 480 (Roche Life Science), including 5 min activation at 95°C and 50 cycles of amplification (10 s at 95°C followed by 30 s at 55°C). The number of 2-LTR circles were normalized for β-actin content.

Mounting of viral particles for fluorescence imaging

$HIV_{IN\text{-}eGFP}$ (1-4 µg of p24 antigen) was immobilized on poly-D-lysine (0.1 mg/ml) coated 8-well chambered cover glasses for 4h at 37°C, washed with PBS and fixed with 4% (v/v) paraformaldehyde. Next, the viral particles were permeabilized with 0.1% (v/v) Triton X-100 (Sigma-Aldrich) in PBS and immunostained for the viral capsid with a monoclonal antibody to p24 (AG3.0, NIH) and secondary goat anti-mouse IgG

Alexa-Fluor 633 conjugate (1/500, Life technologies) diluted in blocking buffer (1% BSA (Sigma-Aldrich) and 0.1% Tween-20 (v/v) (A4974, Applichem) in PBS).

Confocal microscopy

Imaging of cells and viral particles was performed using a laser scanning microscope (Fluoview FV1000, Olympus, Tokyo, Japan). The objective and the excitation polychroic mirror used were UPLSAPO 60x W NA1.2 and DM405/488/559/635, respectively (Olympus). 3D confocal stacks of fixed cells were acquired using a *z*-step size of 0.3 µm and sampling speed of 4 µs/pixel. A 488-nm laser was used for exciting eGFP and Alexa Fluor 633 was excited using a 635-nm diode laser. Emission light was collected at 505-540 and 655-755 nm, respectively. The image resolution was 512 x 512 pixels, a 4x digital zoom and pixel size of 103 nm.

Optimization of image acquisition for intensity measurements

First, to exclude any effect of *z*-position-dependent spherical aberrations on the intensity calculation, 100 nm TetraSpeckTM Microspheres (Invitrogen) were diluted in agarose solution (1 % (w/v) in TAE buffer (UltraPure Low Melting Point Agarose, Invitrogen)) and deposited in 8-well chambered cover glasses, to obtain beads at different *z*-positions. *Z*-stacks were acquired from the glass surface until 15 µm height with a *z*-step of 0.3 µm and the integrated intensity was calculated. Microspheres within a 1.5 µm slice were binned and the mean fluorescence intensity of each bin was calculated. Second, scanning speed and laser power were optimized to obtain the highest signal to noise without pixel saturation for a single IN-eGFP complex.

Quasi-TIRF microscopy and data analysis for FRET measurements

FRET measurements were performed on an inverted microscope (Olympus IX-83, Olympus NV) equipped with a TIRF oil objective. mTFP1 was excited at 445 nm (Cube 445-40c, Coherent, Utrecht, The Netherlands) and mVenus at 514 nm (Sapphire 514-100 CW CDRH, 100 mW, Coherent). The two different laser lines were combined using a dichroic mirror (z488rdc, Chroma Technology GmbH, Olching, Germany). Atto647N was imaged at 644 nm (Excelsior, Newport Spectra Physisc BV, Utrecht, The Netherlands). After combining the laser line with previous laser lines using a dichroic mirror (z532rdc, Chroma Technology GmbH), the laser light is circularly polarized (WPQ05M-532, Thorlabs GmbH, Munich, Germany) and expanded 5 times to achieve a homogenized beam profile in the part of the sample that is imaged on the electron multiplying charge coupled device (EMCCD) chip and passed through a ~1-cm iris to limit the illuminated area in the sample, before finally being focussed on the back focal plane of the objective (PlanApo 60×, NA1.45 oil TIRFM, Olympus) through a 500-mm planoconvex achromatic lens (KPX211-C BK7 Precision Plano-Convex lens, Newport). Emission was collected by the same objective and split by a polychroic mirror (z445/514/633 RPC, Chroma) into a blue emission channel for mTFP1 detection (HQ485/40m, Chroma) and a yellow emission channel for mVenus detection (Razor Edge Long Pass 514 filter, Semrock, New York, USA). Emission of Atto647N was collected by the same objective and split by a 650 long pass dichroic mirror (z650rdc, Chroma Technology GmbH) into a red emission channel. The fluorescence image was expanded 2.5-fold (PE eyepiece 125, 2.5x, Olympus) and focussed onto an EM-CCD (ImagEM, Hamamatsu, Louvain-La-Neuve,

Belgium). The image size was 40×40 µm² and image resolution 512×512 pixels. Images were acquired as the average of 20 consecutive 100 ms integration time frames, and the EM-gain was set on 191.

SUPPLEMENTARY REFERENCES

1. Zack, G.W., Rogers, W.E. & Latt, S.A. Automatic measurement of sister chromatid exchange frequency. *J Histochem Cytochem* **25**, 741-753 (1977).
2. Limpert, E., Stahel, W.A. & Abbt, M. Log-normal distributions across the sciences: Keys and clues. *Bioscience* **51**, 341-352 (2001).
3. Minton, A.P. Influence of macromolecular crowding upon the stability and state of association of proteins: predictions and observations. *J Pharm Sci* **94**, 1668-1675 (2005).
4. Hancock, R. A role for macromolecular crowding effects in the assembly and function of compartments in the nucleus. *J Struct Biol* **146**, 281-290 (2004).
5. Serrao, E. et al. A symmetric region of the HIV-1 integrase dimerization interface is essential for viral replication. *PLoS One* **7**, 10.1371/journal.pone.0045177 (2012).
6. Adachi, A. et al. Production of acquired immunodeficiency syndrome-associated retrovirus in human and nonhuman cells transfected with an infectious molecular clone. *J Virol* **59**, 284-291 (1986).
7. He, J. et al. Human immunodeficiency virus type 1 viral protein R (Vpr) arrests cells in the G2 phase of the cell cycle by inhibiting p34cdc2 activity. *Journal of Virology* **69**, 6705-6711 (1995).
8. Svarovskaia, E.S. et al. Azido-containing diketo acid derivatives inhibit human immunodeficiency virus type 1 integrase in vivo and influence the frequency of deletions at two-long-terminal-repeat-circle junctions. *Journal of Virology* **78**, 3210-3222 (2004).
9. Albanese, A., Arosio, D., Terreni, M. & Cereseto, A. HIV-1 Pre-Integration Complexes Selectively Target Decondensed Chromatin in the Nuclear Periphery. *Plos One* **3**, 2413 (2008).
10. Borrenberghs, D. et al. HIV virions as nanoscopic test tubes for probing oligomerization of the integrase enzyme. *ACS Nano* **8**, 3531-3545 (2014).
11. Chiu, J., March, P.E., Lee, R. & Tillett, D. Site-directed, Ligase-Independent Mutagenesis (SLIM): a single-tube methodology approaching 100% efficiency in 4 h. *Nucleic Acids Research* **32**, e174 (2004).
12. Osorio, L. et al. Viral vectors expressing a single microRNA-based short-hairpin RNA result in potent gene silencing in vitro and in vivo. *J Biotechnol* **169**, 71-81 (2014).
13. Gopaul, D.N., Guo, F. & Van Duyne, G.D. Structure of the Holliday junction intermediate in Cre-loxP site-specific recombination. *Embo J* **17**, 4175-4187 (1998).
14. Kaczmarczyk, S.J., Sitaraman, K., Young, H.A., Hughes, S.H. & Chatterjee, D.K. Protein delivery using engineered virus-like particles. *Proc Natl Acad Sci U S A* **108**, 16998-17003 (2011).
15. Gijsbers, R. et al. LEDGF hybrids efficiently retarget lentiviral integration into heterochromatin. *Mol Ther* **18**, 552-560 (2010).
16. Ibrahimi, A. et al. Highly efficient multicistronic lentiviral vectors with peptide 2A sequences. *Hum Gene Ther* **20**, 845-860 (2009).
17. Salahuddin, S.Z. et al. Restricted expression of human T-cell leukemia--lymphoma virus (HTLV) in transformed human umbilical cord blood lymphocytes. *Virology* **129**, 51-64 (1983).
18. Geraerts, M., Michiels, M., Baekelandt, V., Debyser, Z. & Gijsbers, R. Upscaling of lentiviral vector production by tangential flow filtration. *The Journal of Gene Medicine* **7**, 1299-1310 (2005).
